# The effect of father-child engagement on maternal life satisfaction in South Asian contexts: evidence from MICS surveys in Pakistan and Bangladesh

**DOI:** 10.3389/fsoc.2025.1716756

**Published:** 2026-01-07

**Authors:** Mehr Munir, Anna Bolgrien, Elizabeth Heger Boyle

**Affiliations:** Institute for Social Research and Data Innovation, University of Minnesota Twin Cities, Saint Paul, MN, United States

**Keywords:** father involvement, gender, masculinity, maternal life satisfaction, Multiple Indicator Cluster Survey (MICS), South Asia

## Abstract

Research suggests father involvement in childcare can elevate maternal life satisfaction but has been undertaken almost exclusively in Western countries. This study investigates whether the association holds in countries where gender complementarity is normative, specifically testing the applicability of role theory and family systems theory on mothers’ life satisfaction in under-studied South Asian contexts. We utilize data from Round 6 of UNICEF’s Multiple Indicator Cluster Surveys (MICS), harmonized by IPUMS MICS, for Bangladesh and for three of Pakistan’s provinces. The analytic sample was restricted to married women with 3–4-year-old children (*N* = 34,126). We ran multilevel, mixed-effects logistic regression models for both countries and for Pakistan’s provinces to estimate the relationship between maternal life satisfaction and father’s participation in six different activities (reading or looking at picture books, singing, playing, telling stories, taking child outside, and counting/naming/drawing things). Father reading with child was positively associated with maternal life satisfaction in Bangladesh, and both reading and counting were positively associated in Pakistan’s Sindh province, while storytelling was negatively related in the Khyber Pakhtunkhwa province. Other father activities with children were not significantly associated with maternal life satisfaction. We theorize when fathers perform activities consistent with gender norms, mothers experience higher satisfaction, but when they perform activities that transgress dominant gender norms, women feel uneasy. In conclusion, father-child engagement is sometimes linked to higher maternal life satisfaction in Pakistan and Bangladesh, but this depends on the activity and the context.

## 1 Introduction

Fathers globally are spending an increased amount of time with children within the home, with profound implications for family well-being (Kotila et al., 2013; Pew Research Center, 2013; Dotti Sani and Treas, 2016; Altintas and Sullivan, 2017; Charmes, 2019; Jeong et al., 2023). Whereas fathers were traditionally viewed as financial providers, emergent ideals expand the paternal role to include direct father-child engagement and emphasize father involvement in routine childcare (Offer and Kaplan, 2021). Recent scholarship documents these trends in several cultural contexts including Western, Middle Eastern, and South Asian countries (Seward and Stanley-Stevens, 2014; Sarfaraz et al., 2021).

Evolving fatherhood ideals, coupled with the increasingly recognized need for a more gender-equal division of labor within the home, have spurred substantial research on the impact of father involvement on various individual and family outcomes. Although much of this research focuses on children, an important strand investigates the effect of paternal involvement on maternal life satisfaction (MLS) and well-being. The high prevalence of maternal burnout and anxiety necessitates inquiry into the determinants of MLS, underscoring the significance of this body of research.

However, even though scholarship in the area of father involvement and maternal well-being is growing (Agache et al., 2014; Kim et al., 2016; Nomaguchi et al., 2017; Roskam et al., 2022), its geographical scope is limited. Most scholarship in this field originates from high-income, industrialized nations in the global North, which have limited cultural and socioeconomic overlap with communities in the global South. Societies across the world differ vastly in defining acceptable parameters of male and female behavior; parenting norms are informed by different combinations of value systems, ranging from religion and moral philosophy to individualism and gender egalitarianism (Seward and Stanley-Stevens, 2014; Qian and Sayer, 2016). There is thus a need to investigate the relationship between paternal caregiving and MLS in a variety of contexts, to develop culturally sensitive policies to promote family well-being and improve global maternal and child health. Our study addresses this need by using Multiple Indicator Cluster Surveys (MICS) (UNICEF, 2024), harmonized by IPUMS MICS (Bolgrien et al., 2024) to assess when, whether, and how father-child involvement and MLS are related in two South Asian, lower-middle-income countries—Pakistan and Bangladesh.

Given their unique combination of similarities and differences, a comparative analysis leveraging data from Pakistan and Bangladesh promises valuable insights. The two countries have an intertwined history and similar religious landscapes yet exhibit significant differences in international gender rankings and social markers such as female labor force participation, female literacy, and total fertility rate. In addition to the cross-country comparison, we also conduct a cross-province comparison for the three Pakistani provinces included in our analysis: Punjab, Sindh, and Khyber Pakhtunkhwa (KP).

MICS provides data on six activities that different caregivers, including fathers, may have done with their children in the last three days (reading or looking at picture books, singing, playing, telling stories, taking child outside, and counting/naming/drawing things). Of these activities, three are considered cognitive caregiving (reading, counting/naming/drawing things, telling stories), and three are considered socioemotional caregiving (singing, playing, taking the child outside) (Bornstein and Putnick, 2012). Drawing on role theory and family systems theory, we expect that among these activities, cognitive caregiving will be more strongly associated with MLS than others (as explained below). We also expect to find stronger positive associations between paternal caregiving and MLS in regions with less rigid gender norms, where women would be likely to perceive husband’s support in childcare as incongruent with the dominant masculinity constructs, or as an infringement on her role as a mother. Overall, our analysis provides context-specific and activity-specific insights into the association between father-child engagement and MLS in a world region frequently missing from family research.

## 2 Background

### 2.1 Regional context

Given the important role that social context plays in the relationship between paternal child-related activities and MLS, we now turn to a discussion of the countries and regions studied here. Bangladesh and Pakistan, both among the most populous countries in the world, were part of British India until 1947, and then a single nation until Bangladesh seceded from Pakistan in 1971. In both countries, over 90% of the population is Muslim, which has a profound effect on parenting norms. Islam emphasizes strong family relations and Islamic discourse frequently includes teachings for parents. In addition to Islamic influence, the countries also share influences from other major intellectual and religious traditions of the subcontinent, such as Hinduism. Bangladesh and Pakistan’s similarities provide a strong foundation for comparative analysis. At the same time, the diversity between them, as well as within Pakistan (see, e.g., Table 1), allows an additional layer of insight into how variations in social factors within an overlapping cultural framework affect the association between MLS and father-child engagement.

**Table 1:** Comparison of core indicators across countries and Pakistani provinces.

Core indicator	Country		Pakistani province		
	Bangladesh	Pakistan	Punjab	Sindh	KP
Fertility rate	1.9	3.6	3.4	3.6	4.0
Female literacy	74%	50%	57%	47%	35%
Female labor force participation	37%	25%	31%	18%	19%
Gender gap index (country rank)	24/148	148/148			
Under-5 mortality rate per 1,000 live births	31	58			

Sources: World Bank Open Data, United Nations Population Fund, UN Women, World Economic Forum.

#### 2.1.1 Bangladesh

Bangladesh, with a population of 173 million, is less ethnically diverse than Pakistan; Bengalis are the largest ethnic group (98% of the population) and approximately 99% of the population speaks Bengali, which is the official language of the country. The total fertility rate is notably lower than in Pakistan, at 1.9 births per woman (World Bank, 2022b) (Table 1). Contrasting fertility trends between Pakistan and Bangladesh have been partially attributed to different social and cultural norms, despite the shared majority religion (Lai, 2022). The female literacy rate in Bangladesh is 74%, exceeding the female literacy rate in Pakistan by more than 20 perWcentage points (World Bank, 2022a). Female labor force participation is also higher than in Pakistan but is still fairly low at 37% (World Bank, 2022a, 2022b). Bangladesh’s 2025 ranking on the global gender gap index, compiled by World Economic Forum (WEF), is 24 out of 148 countries, making it the only South Asian country in the top 50 (World Economic Forum, 2025). Caregiving and household labor is highly gendered, with women performing the majority of domestic chores and spending triple the amount of time that men do on housework (Afrin and Saifullah, 2024). Men and women are assigned roles in accordance with patriarchal norms, especially in rural Bangladeshi communities, with men expected to be providers and women expected to be caregivers (Hossain and Atencio, 2017; Islam and Sharma, 2021).

Literature from Bangladesh suggests father involvement in childcare tends to be low but not unusually so. A study conducted in urban slum communities of Bangladesh reported 63% of fathers of children under-two were involved in ensuring adequate nutrition for their child (Bhattacharyya et al., 2023). Research comparing fathers in Bangladesh to fathers in Western countries reports that the former are less likely to avail paternity leave (though the decision is partially financially driven), but when they do take leave, they are more involved in childcare than their American counterparts (Jesmin and Seward, 2011; Murshid, 2016). Younger fathers, especially those living in urban centers, have a more nurturing conception of fatherhood compared to earlier generations, and are more inclined towards engaged and compassionate parenting (Sabur, 2019).

#### 2.1.2 Pakistan

Pakistan consistently features among the poorest-performing countries in the world on gender equality metrics, currently ranking last on the WEF’s gender gap index (World Economic Forum, 2025) (Table 1). Pakistan recently passed the Maternity and Paternity Leave Act of 2023, providing fathers the opportunity to take paid paternity leave, and the country’s total fertility rate of 3.6 births per woman is down from 6.4 births per woman in 2000 (United Nations, 2025). Still, the fertility rate is nearly double that of Bangladesh. Female labor force participation lags at 25%, despite having doubled over the same time period (World Bank, 2018). The vast majority (83%) of women who do not work outside the home cite domestic and childcare responsibilities as their reason for not participating in the labor force (World Bank, 2018), and only half of the female population in Pakistan is literate.

Pakistan is more ethnically and linguistically diverse than Bangladesh, with Punjabis, Pashtuns, Balochis and Sindhis constituting its major ethnic and linguistic groups. Provincial boundaries roughly correspond to the country’s ethnic, linguistic, and cultural differences. In our study, we include three subnational MICS from Pakistan, corresponding to the provinces of Punjab, Sindh, and Khyber Pakhtunkhwa (KP). Each province has a distinct socio-demographic landscape, with different ethnic groups and majority languages.

Development trajectories of Punjab, Sindh, and KP differ significantly from each other, as indicated by their performance on development indicators such as the Human Development Index and Sustainable Development Goals metrics. Punjab typically performs best on development indicators, including women’s education and employment, trailed by Sindh and KP (Georgetown Institute for and Women, Peace and Security, 2022; UN Women, 2023). Female literacy in Punjab, at 57%, is higher than the national average (UN Women, 2023) while its total fertility rate is slightly lower (United Nations Population Fund, 2020). Women’s labor force participation is also highest in Punjab, at 31%, followed by KP at 19% and Sindh at 18%; the national average is 25% (UN Women, 2023). Of the three provinces in this study, KP is the most economically and socio-politically disadvantaged (Rasul and Karki Nepal, 2024; Raza and Pals, 2025).

Importantly, however, these metrics do not fully capture the social constructions of gender that structure women’s lived experiences and perceptions across the Pakistani regions. The provinces are marked by cultural, demographic and linguistic differences, and masculinity in each of the provinces is shaped by distinct socio-political forces. With their historic link to the military, Punjabi (Punjab) and Pakhtun (KP) societies are more likely to embody militarized narratives of “manliness”, which emphasize strength and dominance, and devalue emotional engagement (Rashid, 2022). The legacy of British rule in South Asia, and the British theory of ‘martial races,’ which evolved after the Indian War of Independence in 1857, contribute to the association between manliness and strength/dominance in Punjab and KP. The theory suggested that ethnic and religious groups that remained loyal to the British during the war were noble warriors naturally suited for military service due to inherent qualities like bravery, loyalty, and physical strength (Kakar, 2021). Pakhtuns (also known as Pathans/Pashtuns) and Punjabis were designated as martial races, whereas Sindhis, Memons, and other major ethnicities residing in current Pakistan’s Sindh province were not.

Aspects of this theory continue to resonate in the post-colonial era (Kakar, 2021; Tantray, 2024). This may be particularly true in KP which has been subject to violent military operations and ethnic profiling in the past few decades (Mustafa et al., 2019; Rasul and Karki Nepal, 2024). A recent academic paper authored by professors at a university based in KP provides an illustration that masculinity continues to be linked to militarism in KP: “Men by their prime nature… are harsh, hard, rough, and tough, which compel them to dominate any of the social situations” (Khan et al., 2022). In societies that equate manliness with warrior like qualities, activities like singing/telling stories to young children may be seen as an erosion of manliness and therefore negatively impact spousal happiness.

Recent research on father involvement in the Punjab region suggests a cultural shift in ideas of masculinity there, especially with respect to childrearing practices. In a study conducted with families residing in a rural, low-income community in Punjab, Maselko et al. found that, despite the prevalence of patriarchal norms and traditional gender roles, 72% of fathers were ‘often’ able to help the mother take care of their child (at twelve months old), and 39% played or interacted with the child (Maselko et al., 2019). Additionally, a recent study in urban Punjab on fatherhood ideals found that younger generations more likely to view fathers as nurturing figures compared to preceding generations (Sarfaraz et al., 2021).

In contrast to Punjab and KP, masculinity in Sindh has been relatively less connected to the military, and physical strength, dominance, and ‘toughness’ are not as likely to be equated to masculinity there. However, similar to the rest of Pakistan, earning potential and the breadwinner role is a dominant trait of masculinity in Sindh. The stronger emphasis on men’s economic rather than physical dominance may result in greater acceptability of men’s involvement with young children, especially with respect to cognitive caregiving, which may be seen as a pathway to future economic success.

A qualitative study, performed with an impoverished community in rural Sindh, suggested that although paternal and maternal roles remain deeply gendered (mothers are the primary caregivers and fathers are the providers), father-child engagement can nevertheless fit within the paternal role’s social contours, through activities like taking the child outside, playing with the child, and teaching the child (Jeong et al., 2018).

### 2.2 Theoretical framework

Theories relevant to MLS suggest different, and possibly conflicting ways in which MLS might be related to father’s engagement with children. In this section, we first discuss the mechanisms by which father’s involvement in child-centered activities might be expected to enhance MLS. We then discuss reasons to expect no relationship or even a negative relationship between the two phenomena based on gender expectations in particular social contexts. Additionally, we lay out expectations for the type of activities more likely to be positively associated with MLS in the study populations included in our analysis. We conclude this section with a discussion of the different hypotheses our theoretical reasoning gives rise to, and which we then proceed to test.

#### 2.2.1 Theoretical frames suggesting a positive association between MLS and father-child engagement

Role theory is a commonly invoked framework to understand and articulate the stressors associated with parenthood (Ventura, 1987; Petts and Knoester, 2019). Roles refer to the demands the social system places on a person in a specific social position, and to the behaviors it expects of that person (Duxbury et al., 2008). Role overload describes situations in which the tasks and responsibilities attached to the roles a person performs exceed their capacity to fulfill them (McIntosh et al., 2024), and is associated with depression, lower relationship quality, and time poverty (Perry-Jenkins et al., 2007). Mothers of young children, ranging from infants to adolescents, are particularly susceptible to role overload, given the intense demands of caring for young children combined with gendered parenting norms that assign childcare responsibility primarily to the mother (Perry-Jenkins et al., 2007; Helms et al., 2010; Ruppanner et al., 2019). Across the globe, mothers spend significantly more time caring for children than fathers, and engage in more routine and repetitive tasks (Craig, 2006; Craig and Mullan, 2011; Offer, 2014; McDonnell et al., 2019). Additionally, they experience higher mental load performing most of the emotional and cognitive labor associated with parenting (Offer and Schneider, 2011; Robertson et al., 2019; Dean et al., 2022; Haupt and Gelbgiser, 2024). Father involvement in childcare has the potential to diminish role overload, and in that way can elevate MLS.

Beyond role theory, family systems theory is also relevant to the current analysis. Family systems theory stresses the interconnectedness of members and relationships within the family and highlights the reciprocity of the “spousal subsystem” and the “parental subsystem” (Galovan et al., 2014). From this perspective, successful father-child relationships exert a positive influence on the family as a whole and should increase MLS across social contexts. Additionally, if father-child engagement leads to increased well-being for the father and/or the child as individuals, that would also be a pathway for higher MLS (Chopik and O’Brien, 2017; Cabrera, 2020).

Several studies from wealthy countries support these theories, finding an association between fathers’ involvement with children and positive outcomes for mothers. For example, an analysis that used data from the United States-based Fragile Families and Child Wellbeing study reported a negative association between father involvement in childcare for children under five and maternal parenting stress (Nomaguchi et al., 2017). Another study conducted with the same dataset did not find this association, but found that emotional parenting support by the father alleviated maternal parenting stress (Harmon and Perry, 2011). In an analysis that deployed data from the German Socio-Economic Panel, Agache et al. (2014) reported higher father involvement in the first three years after childbirth was associated with higher life satisfaction for both parents. Utilizing the same dataset, Preisner et al. (2020) demonstrated MLS in Germany is higher than in preceding decades, which the authors attributed to more gender-egalitarian parenting and lower prevalence of gendered division of domestic labor. Petts and Knoester (2019) also report positive associations between paternity leave-taking and MLS and/or relationship satisfaction. These results are significant from both the role overload and family systems perspective, suggesting that paternal caregiving enhances MLS not only by mitigating mother’s role overload but also through elevated paternal well-being.

Research from Asian countries, which mostly focuses on East Asian countries, also suggests a relationship between father-child engagement and MLS (Kasamatsu et al., 2021; Lee, 2024; Song et al., 2024). A nation-wide study of Japanese families with children under one showed the father activity that was most strongly associated with maternal well-being was playing with the child at home, suggesting stronger support for family systems theory than role overload (Kasamatsu et al., 2021). In a study of Korean families, Kramer et al. (2019) used nationally representative longitudinal data to examine the pathways through which paternity leave might benefit families, specifically delving into MLS and relationship satisfaction, among other outcomes. The authors did not find a direct relationship between paternity leave and MLS but did find an indirect relationship among couples who agreed on gender roles (more engaged fathers in these couples tended to be happier and their greater happiness was associated with higher MLS). In a separate study that used longitudinal data from Singapore to investigate the impact of father’s paternity leave, the authors found that paternity leave of two weeks or longer reduced mother’s parenting aggravation, defined as feelings of exhaustion, feeling burdened, and feeling trapped (Yeung and Li, 2023).

#### 2.2.2 Theoretical frames suggesting absent or negative association between MLS and father-child engagement

Some research specifically challenges the universality of a positive relationship between father’s engagement with young children and mother’s well-being, and suggests this relationship is context specific. In societies that emphasize gender complementarity, or the idea that men and women serve distinct roles in the household, fathers providing childcare may challenge notions of masculinity (Magaraggia, 2013; Peukert, 2019). When traditional gender norms dominate, and mothering and domesticity are cornerstones of femininity, father-child engagement may lead to *role conflict* in terms of expectations regarding husbands’ masculinity and the desire for help with childrearing (Magaraggia, 2013; Peukert, 2019). These conflicted feelings may result in father-child engagement being unassociated with MLS, or even negatively associated. If child engagement and caregiving are considered maternal and feminine, and ‘masculine’ fathers are expected to remain aloof and focus on financial provision, regular father-child engagement may be perceived as superfluous or aberrant.

These ideas were supported in the findings of a study by Roskam et al. (2022) of “burnout” among mothers across 41 countries. The study included families with at least one child (of any age) living at home, and determined the level of burnout based on women’s responses to survey questions suggesting emotional exhaustion, loss of pleasure in parenting, and emotional distancing from children. Roskam et al. found higher levels of burnout among mothers lacking childcare support from their spouses when those women lived in countries with structures *promoting* gender equality in education, employment, and political participation, and who personally held gender-egalitarian attitudes. The researchers attributed this surprising association to a gap in expectations when gender equality outside the home is not mirrored in the marital relationship within the home. They also suggested more egalitarian gender norms within societies make it more likely women will compare themselves to their spouses, but complementary gender norms make it more likely women will use only other mothers as their reference group. The implication for societies with conservative gender norms is that women’s satisfaction may be less tied to fathers’ activities with children because women have little or no expectation that their husbands will provide support in this way.

Another study that highlights the influence of gender role attitudes among American couples reported longer paternity leave was associated negatively with maternal reports of relationship conflict among working mothers, but positively among mothers who did not work (Petts and Knoester, 2019). The authors speculated mothers who were not in the paid labor force may have perceived father’s presence in the domestic space as problematic or threatening and/or may have had higher standards for domestic work than employed mothers, which the fathers were unable to meet.

### 2.3 Hypotheses

#### 2.3.1 Hypotheses about child-engagement activities

Because educational pursuit is a favorable norm for men in both Pakistan and Bangladesh, fathers engaging children in cognitive activities—particularly reading and counting—is likely compatible with gender expectations in both countries. For this reason, we expect mothers will perceive less role conflict with father activities related to reading or counting versus other activities and consequently derive a greater sense of satisfaction from those activities versus socioemotional activities. This may be especially true in Pakistan—because women tend to have low levels of education there, there may be a stronger expectation husbands will do the work of preparing children for school. Despite Pakistan’s low national literacy rate, school enrollment for their children is highly desired by both parents (Ahmed et al., 2024).

#### 2.3.2 Hypotheses about varying social contexts

We anticipate that the association between MLS and father engagement will vary across national and provincial contexts, as cultural norms surrounding marriage and gender will moderate this association. Due to conservative gender norms and traditional masculinity constructs, the benefits of family strengthening and role alleviation may be offset by the social costs of violating these norms and failing to conform to expected gender roles, particularly in more patriarchal settings. With Bangladesh’s relatively higher degree of gender equality and greater integration of women in the public sphere, we speculate the conflict between father-child engagement and social norms will be lower there, resulting in positive associations. Within Pakistan, we anticipate stronger positive associations in Sindh province, despite its low performance on female empowerment indicators, since it is not characterized by militarized constructs of masculinity like the other provinces in our analysis. Another possibility, however, is that women in the Punjab region of Pakistan, which has shown more improvement on quantitative gender metrics than either Sindh or KP, will be more positively affected by fathers’ engagement than mothers in Sindh and KP.

## 3 Materials and methods

### 3.1 Data

Our data comes from Round 6 of the Multiple Indicator Cluster Surveys (MICS), accessed through IPUMS MICS. MICS is an international household survey series, developed and supported by UNICEF since the 1990s. With a strong focus on women and children, MICS is a leading source of data on global maternal and child health. The surveys employ multistage probability designs to ensure nationally or subnationally representative samples (Khan and Hancioglu, 2019). For selected households, data are obtained on all women aged 15–49 and all children aged 0–4 using the women and the children’s questionnaire forms, respectively. Response rates for MICS typically exceed 90% (Khan and Hancioglu, 2019).

IPUMS MICS automates harmonization of MICS data through providing the required syntax; it also simplifies the process of dataset construction by enabling customized data extracts. Due to consistent coding of variables across time and space and ready availability of relevant metadata, IPUMS MICS is particularly well-suited for cross-national research.

Our dataset comprised three samples from Pakistan, for the provinces of Punjab (Pakistan Bureau of Statistics and UNICEF, 2019b), Sindh (Pakistan Bureau of Statistics and UNICEF, 2019c), and KP (Pakistan Bureau of Statistics and UNICEF, 2019a) and one sample from Bangladesh (Bangladesh Bureau of Statistics and UNICEF, 2019), collected between 2017 and 2019. The Bangladesh sample is nationally representative, and each Pakistan sample is representative at the provincial level. In our main analyses, we pooled data from the Pakistan samples and produced country-specific descriptive statistics and regression models. In supplementary analyses, we examined each Pakistan provincial sample separately.

Data for this analysis consisted of women aged 15 to 49 with 3–4 year old children, who were married and lived in the same household as their child’s biological father. For women with multiple children aged 3–4 years, we retained data only for the youngest child (in the case of twins and other multiple births, only one child is included). The number of women eligible for inclusion in our analysis was 35,467, from which we excluded observations missing the dependent variable (*n* = 82), the paternal engagement variables (*n* = 131), or control variables (*n* = 1,128).^1^ In the final dataset (*N* = 34,126), each observation corresponded to an individual woman with characteristics of her child and spouse, including indicators of father-child engagement, attached to her record.

### 3.2 Measures

Our dependent variable was self-reported life satisfaction. All women respondents were asked the question, “taking all things together, would you say you are very happy, somewhat happy, neither happy nor unhappy, somewhat unhappy or very unhappy?” Response options ranged from 1 = “very happy” to 5 = “very unhappy”. A figure consisting of smiley faces with expressions ranging from happy to sad was shown to respondents to facilitate interpretation of the question. Due to the skewed distribution of responses, we recoded life satisfaction into a binary outcome: respondents reporting “neutral,” “somewhat unhappy,” or “very unhappy” were coded as 0 (“not satisfied”), while those reporting “somewhat happy” or “very happy” were coded as 1 (“satisfied”), as done by other studies (Yaya et al., 2019; Ujah et al., 2024).

Paternal engagement variables were taken from the MICS module on early childhood development, which is part of the questionnaire for children under five, and is typically administered to the child’s mother. The child’s mother reports for herself, the child’s father, and any non-parent adult in the household, whether or not they participated in each of the following six activities with the child in the past three days: reading or looking at picture books, singing, playing, telling stories, taking child outside, and counting/naming/drawing things. In our dataset, we have binary variables for whether or not the father performed each activity.

To minimize risk of confounding, we controlled for child, mother, father, and household characteristics that could influence MLS or paternal engagement. Child controls included child sex and birth order. We controlled for mother’s age and education and – because they may affect her life satisfaction – her attitude toward domestic violence, whether she had any male children, whether she had a child who died, and total number of biological children under 5 (to account for the presence of infants in the mother’s care in addition to the 3–4 year old child this analysis is centered on). We also controlled for father’s education. At the household level, we included variables for household wealth, urban/rural status, and a binary for whether an adult other than the parents engaged in any of the six activities with the child in the last three days. Additionally, we added a ‘maternal engagement score’ variable, which was created by summing the number of caregiver activities the mother reported doing with the child in the past three days.

### 3.3 Analytic strategy

All statistical analyses were conducted using Stata 18. To account for the complex survey design, we applied survey weights in the descriptive analysis, using the svyset suite of commands. To estimate the relationship between MLS and paternal engagement, we implemented a series of multilevel, mixed-effects logistic regression models (melogit in Stata). Multilevel modeling was appropriate given the hierarchical structure of the data, and the potential for ecological effects due to varying social norms. We utilized two-level models, with individuals at the first level and the community (proxied by district) at the second level. In Pakistan, districts are the third administrative level; whereas smaller cities collectively comprise one district, big cities like Karachi have multiple districts. In Bangladesh, districts are the second level of geography. Random effects at the community level were incorporated to account for unobserved heterogeneity between communities to address regional variations. All models were country-specific or sample-specific, and results are presented separately for Pakistan and Bangladesh.

For each sample and for the Pakistani samples combined, we ran each model with one father engagement variable at a time (reading or looking at picture books, singing, playing, telling stories, taking child outside, or counting/naming/drawing things) and the control variables. The binary dependent variable was consistent across all models. After the country-level models, we ran province-level models using data from the Pakistan samples only.

## 4 Results

### 4.1 Descriptive analysis results

Characteristics of the study sample are provided in Table 2. Compared to Bangladesh, women in the Pakistan samples had more children on average (4.0 versus 2.4). Accordingly, a larger proportion of children in the Pakistan samples were of fourth or higher birth order (37.2% versus 12.5%), the mothers were older, on average (31.3 years versus 28.5 years), and household sizes were also larger (8.7 versus 5.3). Support for domestic violence appeared high in both countries, but was considerably higher in Pakistan, with 42.7% women justifying domestic violence in some situations, compared to 28.9% in Bangladesh. Additionally, educational profiles of the samples differed significantly; most women in Bangladesh possessed secondary education (48.1%), but most women in Pakistan had less than primary education (56.5%). Notably, the proportion of men who had secondary education or higher in Bangladesh (46.5%) was lower than the proportion of women who had secondary education or higher (62.4%). In Pakistan, the reverse was true; whereas 52.2% of men had secondary education or higher, only 28.4% of women possessed secondary education or higher.

**Table 2:** Descriptive statistics for women, children, and households included in the analysis, by country and province.

Variable	Country Mean (SD) or *n* (%)		Pakistani province Mean (SD) or *n* (%)		
	Bangladesh (*n* = 7,644)	Pakistan (*n* = 26,109)	Punjab (*n* = 12,827)	Sindh (*n* = 6,033)	Khyber Pakhtunkhwa (*n* = 7,250)
Respondent characteristics					
Age (mean, SD)	28.509 (5.9)	31.300 (6.1)	31.504 (5.8)	31.345 (6.5)	30.904 (6.4)
Education					
Less than primary	952 (12.4)	14,743 (56.5)	5,762 (44.9)	3,929 (65.1)	5,052 (69.7)
Primary	1,921 (25.1)	3,943 (15.1)	2,495 (19.5)	692 (11.5)	757 (10.4)
Secondary	3,680 (48.1)	4,702 (18.0)	2,969 (23.1)	832 (13.8)	900 (12.4)
College	1,092 (14.3)	2,721 (10.4)	1,600 (12.5)	580 (9.6)	542 (7.5)
Number of surviving children (mean, SD)	2.4 (1.3)	4.0 (2.0)	3.9 (1.9)	3.9 (2.1)	4.1 (2.1)
Domestic violence justification^1^					
Yes	2,212 (28.9)	11,147 (42.7)	3,946 (30.8)	2,370 (39.3)	4,830 (66.6)
Self-reported happiness					
Very unhappy	79 (1.0)	247 (0.9)	126 (1.0)	42 (0.7)	79 (1.1)
Somewhat unhappy	136 (1.8)	725 (2.8)	342 (2.7)	167 (2.8)	216 (3.0)
Neither happy nor unhappy	832 (10.9)	3,647 (14.0)	1,906 (14.9)	623 (10.3)	1,118 (15.4)
Somewhat happy	4,696 (61.4)	10,888 (41.7)	4,929 (38.4)	2,894 (48.0)	3,065 (42.3)
Very happy	1,900 (24.9)	10,602 (40.6)	5,523 (43.1)	2,306 (38.2)	2,772 (38.2)
Child characteristics					
Age (years)					
Three	3,960 (51.8)	14,453 (55.4)	7,109 (55.4)	3,273 (54.3)	4,071 (56.2)
Four	3,685 (48.2)	11,656 (44.6)	5,717 (44.6)	2,760 (45.7)	3,179 (43.8)
Sex					
Female	3,663 (47.9)	12,447 (47.7)	6,215 (48.5)	2,815 (46.7)	3,417 (47.1)
Birth order					
First	2,721 (35.6)	6,007 (23.0)	2,935 (22.9)	1,477 (24.5)	1,595 (22.0)
Second to third	3,965 (51.9)	10,379 (39.8)	5,377 (41.9)	2,349 (38.9)	2,653 (36.6)
Fourth to sixth	889 (11.6)	7,661 (29.3)	3,700 (28.8)	1,671 (27.7)	2,290 (31.6)
Seventh or higher	68 (0.9)	2,062 (7.9)	815 (6.4)	535 (8.9)	712 (9.8)
Paternal and household characteristics					
Father’s education					
Less than primary	1,733 (22.7)	8,225 (31.5)	3,311 (25.8)	2,465 (40.9)	2,450 (33.8)
Primary	2,360 (30.9)	4,236 (16.2)	2,490 (19.4)	889 (14.7)	858 (11.8)
Secondary	2,308 (30.2)	9,202 (35.2)	5,149 (40.1)	1,475 (24.4)	2,578 (35.6)
College	1,243 (16.3)	4,446 (17.0)	1,878 (14.6)	1,204 (20.0)	1,364 (18.8)
Household size (mean, SD)	5.258 (2.1)	8.713 (4.503)	8.191 (4.091)	8.380 (4.342)	9.912 (5.069)
Urban status					
Urban	1,701 (22.2)	8,480 (32.5)	4,469 (34.8)	2,809 (46.6)	1,202 (16.6)
Wealth index quintile					
Poorest	1,801 (23.6)	6,340 (24.3)	3,088 (24.1)	1,581 (26.2)	1,671 (23.0)
Second poorest	1,623 (21.2)	5,352 (20.5)	2,584 (20.1)	1,304 (21.6)	1,464 (20.2)
Middle	1,408 (18.4)	4,934 (18.9)	2,430 (18.9)	1,183 (19.6)	1,321 (18.2)
Second richest	1,391 (18.2)	4,945 (18.9)	2,460 (19.2)	1,099 (18.2)	1,386 (19.1)
Richest	1,420 (18.6)	4,538 (17.4)	2,263 (17.6)	866 (14.4)	1,409 (19.4)
Child-engagement^2^					
Father read books to child	1,482 (19.4)	1,949 (7.5)	749 (5.8)	730 (12.1)	470 (6.5)
Father sang songs to child	1,089 (14.3)	1,849 (7.1)	602 (4.7)	913 (15.1)	334 (4.6)
Father took child outside	3,149 (41.2)	12,055 (46.2)	4,836 (37.7)	3,677 (60.9)	3,543 (48.9)
Father played with child	1,100 (14.4)	3,979 (15.2)	1,725 (13.5)	1,240 (20.5)	1,015 (14.0)
Father told stories to child	1,401 (18.3)	2,375 (9.1)	834 (6.5)	941 (15.6)	599 (8.3)
Father spent time with child counting/naming/drawing things	1,329 (17.4)	1,488 (5.7)	622 (4.8)	481 (8.0)	385 (5.3)
Child engaged by a non-parent figure in the household	3,926 (51.4)	14,860 (56.9)	6,095 (47.5)	4,081 (67.7)	4,684 (64.6)

All values are weighted in accordance with the survey design. For categorical variables, we report frequencies with percentages in parentheses; for continuous variables, we report means with standard deviations (SD) in parentheses. The respondent was asked if wife-beating is justified in any of the following circumstances: if she goes out without telling her husband, neglects the children, burns the food, argues with her husband, or refuses sex. 1. The respondent was asked if wife-beating is justified in any of the following circumstances: if she goes out without telling her husband, neglects the children, burns the food, argues with her husband, or refuses sex. 2. All child engagement variables refer to activities performed in the last three days.

Self-reported life satisfaction was high in both countries, with approximately 86.3% women in Bangladesh describing themselves as ‘very happy’ or ‘somewhat happy’ in Bangladesh, and approximately 82.3% women doing the same in Pakistan. A larger proportion of women were in the ‘very happy’ category in Pakistan (40.6% versus 24.9%).

In both countries, the most commonly reported activity performed by the child’s father was taking the child outside (41.2% of fathers in the Bangladesh sample took child outside in the last three days, and 46.2% of fathers in the combined Pakistan samples did the same). Reading to the child was the second most common activity in Bangladesh (19.4% of fathers in the Bangladesh sample did so), and playing with the child was the second most common in Pakistan (15.2% of fathers in the combined Pakistan samples did so). The activity with the lowest paternal participation in Bangladesh was singing to the child (14.3%), but in Pakistan the activity with the lowest paternal participation was counting/naming/drawing things with the child (5.7%). It is noteworthy that in Bangladesh, none of the activities had lower than 10% paternal participation, but in Pakistan, four of the six activities had less than 10% paternal participation.

Descriptive statistics at the provincial level illustrate considerable variation in socio-demographic characteristics and father-engagement patterns within Pakistan. Women from KP had the highest number of children on average (4.1 versus 3.9 in Punjab and Sindh) and were slightly younger compared to their Sindhi and Punjabi counterparts (30.9 years versus 31.3 and 31.5 years, respectively). The proportion of women who had at least primary education was highest in Punjab, followed by Sindh and then KP. Support for domestic violence was strikingly high in KP, at 66.6%; in Punjab and Sindh it was 30.8 and 39.3%, respectively. Sindh had the highest percentage of women who were in either the ‘somewhat happy’ or ‘very happy’ category (86.2%), followed by Punjab (81.5%) and KP (80.5%), although Punjab had the highest percent of women in the ‘very happy’ category (43.1% versus 38.2% in both Sindh and KP). Taking the child outside was the most frequently reported paternal activity in all three provinces, and playing with the child was the second most frequent. Singing to the child was the least common activity in both Punjab and KP, but counting/naming/drawing things was the least common in Sindh.

### 4.2 Results of logistic regression analysis

Socio-demographic controls related to wealth and education yielded expected and similar results in both countries (full tables with control variables included are provided in the Appendix). Additionally, support for domestic violence was significantly negatively associated with life satisfaction in both Pakistan and Bangladesh. Female sex of the child was positively associated with MLS in Bangladesh, but negatively in Pakistan. Higher birth order of the child was also significantly negatively associated with MLS in Pakistan.

Results from multilevel logistic regressions for each type of father-child development activity are presented in Table 3. Each cell in represents a separate regression equation, as the models were run separately for the activities and for each country and province. Full results showing control variable associations are provided in the Appendix. In both Bangladesh and Pakistan, MLS was associated with some types of father-child engagement but not all. In Bangladesh, father’s reading (OR = 1.28, *p* < 0.05) was significantly positively related to an increase in mother’s reported life satisfaction. For Pakistan, reading (OR = 1.25, *p* < 0.01) and counting/naming/drawings things (OR = 1.23, *p* < 0.05) was significantly positively related to mother’s life satisfaction.

**Table 3:** Father-child engagement association with mothers’ life satisfaction, by country and Pakistani province in multilevel logistic regressions.

Child engagement activity	Country		Pakistani province		
	Bangladesh	Pakistan	Punjab	Sindh	Khyber Pakhtunkhwa
	(*n* = 7,754)	(*n* = 26,372)	(*n* = 12,824)	(*n* = 6,226)	(*n* = 7,322)
	OR (95% CI)	OR (95% CI)	OR (95% CI)	OR (95% CI)	OR (95% CI)
Father read to child	1.28* (1.03–1.59)	1.25** (1.06–1.47)	1.12 (0.86–1.46)	2.28** (1.64–3.17)	0.91 (0.69–1.20)
Father sang to child	1.09 (0.86–1.38)	1.00 (0.87–1.16)	1.18 (0.90–1.55)	1.16 (0.91–1.48)	0.77* (0.59–1.00)
Father played with child	0.99 (0.79–1.24)	1.02 (0.92–1.14)	1.04 (0.88–1.22)	0.84 (0.67–1.04)	1.18 (0.98–1.42)
Father took child outside	1.14 (0.97–1.33)	0.99 (0.92–1.07)	1.02 (0.92–1.13)	0.87 (0.73–1.04)	1.05 (0.92–1.20)
Father told stories to child	1.19 (0.96–1.48)	0.96 (0.84–1.09)	0.99 (0.78–1.25)	1.25 (0.96–1.61)	0.79* (0.64–0.99)
Father counted, named, or drew things with child	1.22 (0.97–1.52)	1.23* (1.03–1.46)	1.24 (0.93–1.65)	1.48* (1.06–2.08)	1.12 (0.83–1.51)

Models were run separately for each country and region for each activity. Controls for all models (not shown) included mother’s age and education, total number of children under 5 (biological), child’s sex, child’s birth order, father’s education, wealth index quintile, urban status, any male children, any deceased children, mother’s endorsement of domestic violence, if anyone else in household engaged child in the last three days, and mother’s engagement score. Full regression results are provided in the Appendix. *p < 0.05; **p < 0.01.

Table 3 also illustrates the association between MLS and each of the six father-child activities separately for Punjab, Sindh, and KP. In Punjab, five of the six activities were positively associated with MLS, but none was significant. In Sindh, four of the six activities were significantly positively associated with MLS, and two were significant – reading (OR = 2.28, *p* < 0.01) and counting/naming/drawing things (OR = 1.48, *p* < 0.05). In KP, three activities were positively associated with MLS but were insignificant. Telling stories to the child, however, was significantly negatively associated with MLS (OR = 0.79, *p* < 0.05), while singing to the child was also negative and borderline significant (OR = 0.77, *p* < 0.05).

## 5 Discussion

Our results suggest only certain types of father engagement with children (reading and counting) are associated with the well-being of mothers in the study populations, and only in some contexts. Additionally, our findings highlight the limitations of applying aggregated data to subnational regions, as well as the inadequacy of quantitative gender indices in capturing the complexities of gender realities on the ground.

In both Bangladesh and Pakistan, fathers reading to their children was positively associated with MLS, and, in Pakistan, fathers counting/naming/drawing things with their children was also positively associated with MLS. Thus, mothers seem to particularly value father’s participation in activities generally categorized as cognitive caregiving. Socioemotional caregiving activities were not associated with MLS in either country and, in the KP region of Pakistan, some of these activities (storytelling and singing) were negatively associated with MLS.

Firstly, from the perspective of gender norms, paternal engagement in cognitive caregiving may be more readily acceptable in societies with traditional gender constructions, compared to socio-emotional caregiving activities. Teaching a child to read and count, for example, may be considered important activities that neither challenge masculinity nor undermine father’s status as head of household, in contrast to mundane tasks or more playful types of child-engagement. On the other hand, activities like singing to the child may be viewed as too feminine and unbecoming of the head of the household in societies where strict gender norms prevail.

From a family systems perspective, cognitive caregiving may be especially impactful as research suggests in resource-constrained settings, socioemotional caregiving is more readily performed by all caregivers (including mothers, fathers, and non-parental figures), compared to cognitive caregiving (Rothenberg and Bornstein, 2023). Therefore, in lower- and middle-income country settings, children from families in which the father cognitively engages the child would be more likely to thrive, exerting a positive influence on the family unit. Considering the results from a role overload perspective, it is plausible that mothers place greater value on fathers’ engagement in activities like reading and counting/naming/drawing things with the child, as these activities are more time-consuming and typically involve one-on-one interaction. This type of father-child engagement may therefore free up more of the mother’s time and energy. Additionally, given the low levels of female education and literacy in Pakistan, women may find it difficult to engage with the child on these things themselves and may therefore especially appreciate the father’s involvement.

Expanding the lens to include subnational regions, we see the positive associations among father activities and MLS in Pakistan were driven largely by Sindh province. Our descriptive results indicate higher father involvement in Sindh than either KP or Punjab. In addition, regression analyses showed no statistically significant positive relationships between father engagement and MLS in either Punjab or KP. Although null results in Punjab are surprising, the strong association of mother’s life satisfaction with father-child engagement in Sindh is consistent with our knowledge of cultural differences in the meaning of masculinity across Pakistani regions. Due to the absence of military-related influences on the construction of masculinity in Sindh, there is likely greater acceptance of men’s involvement with young children, especially for cognitive caregiving activities. Like Bangladesh, Sindh seems to embody gender norms that allow men to engage with young children, whereas in Punjab and KP, child-engagement is potentially at odds with masculinity. Conversely, women in Punjab and KP may feel content satisfaction carrying out their assigned mothering role within clear gender demarcations, and a partner who frequently engages with children may be seen as breaching masculinity, potentially offsetting the benefits from role alleviation.

Importantly, in the KP region, we not only failed to find positive associations between father engagement and MLS, we discerned statistically significant negative associations for storytelling and singing. This may be linked to recent armed conflict in KP, which could be perpetuating historic links between manliness and being “harsh, hard, rough, and tough” (Khan et al., 2022). In this context, socioemotional activities by fathers could call their masculinity into question, negatively impacting spousal happiness. KP also has the lowest levels of female education and literacy among the three regions of Pakistan studied. It may be that, because overall education is low in KP, and schooling opportunities remain limited, involving a child in reading or similar activities is not considered useful.

Overall, our results are consistent with how masculinity is viewed in Sindhi versus Punjabi and Pakhtun societies, leading us to theorize cultural variations in the construction and enactment of masculinity matter more than women’s empowerment metrics in understanding the relationship between father-child engagement and MLS. If quantitative markers of female empowerment were driving our findings, we would have found positive associations between father engagement and MLS in Punjab, which tends to score highest on such measures. These findings illustrate the significance of social factors that are difficult to quantify, and may be missed by standard global metrics of women’s empowerment, in shaping the lives of women and their families.

Other noteworthy results include the strong and significant negative relationship between supportive attitudes towards domestic violence and women’s life satisfaction in both countries and every region. These results indicate that women who accept gender-based power differentials and endorse partner-inflicted violence have lower life satisfaction than women who reject any justification for domestic violence. For women who support domestic violence, perceived subservience to men likely undermines life satisfaction through multiple pathways (Dillon et al., 2013). At the same time, however, widespread supportive attitudes towards domestic violence, like in KP, likely reflect specific cultural constructions of gender that render father-child engagement irrelevant or undesirable. In such contexts, the presence or absence of father-child engagement may be unrelated or negatively to women’s life satisfaction, until the relationship between ideas of masculinity and violence is eroded.

## 6 Conclusion

Father-child engagement is under-studied in non-Western societies, despite its potential to enhance family well-being and improve maternal and child health. As family structures and gender norms evolve at an unprecedented pace across the globe, there is a growing need for diverse scholarly inquiry into how new fatherhood ideals are manifesting across different cultures and interacting with maternal experiences in varied contexts. Our study contributes by exploring paternal engagement as a correlate of MLS in cultural contexts frequently missing from global family research. The cross-country and cross-province comparisons included in our analysis generate nuanced insights into a region rarely studied at a granular level and highlight the complexity of the socio-cultural factors that shape MLS.

Our overall assessment is although social context moderates the association between MLS and father engagement, a positive relationship with cognitive caregiving activities is likely to hold across a wide range of societies. Expanding the role of fathers in child-engagement and upbringing in culturally informed ways is likely to yield benefits across social contexts and should be an important consideration in policies around employment, family life, and public health. Additionally, normalizing father-child engagement through reimagined representations of masculinity in mass media, and highlighting fathers in public health and educational campaigns, may further enhance the potential of paternal involvement to elevate MLS.

## 7 Limitations

Among the key limitations of our analysis is unavailability of respondent’s employment status. Availability of employment information would have strengthened our evaluation of how well role theory applies in the regions included in our analysis. Employment status and decision-making power variables may have yielded insight into the effect of female empowerment as well, but are not included in MICS. Additionally, we do not have data on the duration of father-child engagement, which could be an important factor. Finally, because we are analyzing survey data, we cannot establish causality.

## Data Availability

Publicly available datasets were analyzed in this study. This data can be found at: https://mics.unicef.org/surveys.

## Footnotes

1 Sensitivity analyses suggested no meaningful differences between women who were dropped due to missing values on the key variables, and women who were retained in the sample. Additionally, missing observations were distributed across a multitude of variables, mitigating concerns of bias.
