# Supplementary material for: The effect of father-child engagement on maternal life satisfaction in South Asian contexts: evidence from MICS surveys in Pakistan and Bangladesh
Source: Front Sociol. 2026 Jan 7;10:1716756. doi: 10.3389/fsoc.2025.1716756 (PMC12819588; doi:10.3389/fsoc.2025.1716756)
Supplement: Supplementary file 1 [file Data_Sheet_1.PDF]

Appendix

Table A1. A*ssociation between father reading to child in the last 3 days and mothers' life satisfaction, by country and Pakistani province in multilevel logistic regressions.*

	**Country**				**Pakistani Province**					
	**Bangladesh** (n=7,754)		**Pakistan** (n= 26,372)		Punjab (n=12,824)		Sindh (n=6,226)		Khyber Pakhtunkhwa (n= 7,322)	
	OR	(95% CI)	OR	(95% CI)	OR	(95% CI)	OR	(95% CI)	OR	(95% CI)
Father read to child	1.28*	(1.03 - 1.59)	1.25**	(1.06 - 1.47)	1.12	(0.86 - 1.46)	2.28**	(1.64 - 3.17)	0.91	(0.69 - 1.20)
Child is female	1.20*	(1.00 - 1.43)	0.91**	(0.85 - 0.97)	0.89*	(0.80 - 0.98)	1.00	(0.84 - 1.18)	0.90	(0.79 - 1.03)
Child's birth order	0.87	(0.75 - 1.01)	0.87**	(0.82 - 0.91)	0.89**	(0.83 - 0.96)	0.78**	(0.70 - 0.88)	0.89*	(0.81 - 0.98)
Mother age	1.00	(0.99 - 1.02)	0.99**	(0.98 - 1.00)	0.98**	(0.97 - 0.99)	1.00	(0.99 - 1.02)	0.99	(0.98 - 1.00)
Mother education (ref. = less than primary)										
Primary	1.47**	(1.20 - 1.80)	1.04	(0.93 - 1.15)	1.01	(0.88 - 1.15)	1.09	(0.83 - 1.44)	0.99	(0.78 - 1.25)
Secondary	2.05**	(1.64 - 2.57)	1.17*	(1.03 - 1.32)	1.08	(0.92 - 1.26)	0.97	(0.70 - 1.34)	1.32*	(1.02 - 1.70)
College	2.15**	(1.43 - 3.22)	1.30**	(1.08 - 1.56)	1.18	(0.92 - 1.51)	1.16	(0.73 - 1.85)	1.38	(0.97 - 1.96)
Father education (ref. = less than primary)										
Primary	1.16	(0.97 - 1.39)	1.15**	(1.05 - 1.27)	1.26**	(1.11 - 1.43)	1.14	(0.91 - 1.43)	1.05	(0.85 - 1.29)
Secondary	1.31*	(1.06 - 1.61)	1.29**	(1.18 - 1.41)	1.54**	(1.36 - 1.74)	1.04	(0.83 - 1.30)	1.10	(0.94 - 1.28)
College	2.49**	(1.67 - 3.71)	1.61**	(1.41 - 1.84)	2.10**	(1.70 - 2.60)	1.54**	(1.15 - 2.06)	1.26*	(1.02 - 1.57)
Wealth quintile (ref. = middle)										
Poorest	0.48**	(0.39 - 0.60)	0.56**	(0.50 - 0.62)	0.49**	(0.41 - 0.57)	0.45**	(0.35 - 0.58)	0.80*	(0.65 - 0.97)
Second poorest	0.70**	(0.55 - 0.88)	0.79**	(0.71 - 0.88)	0.72**	(0.62 - 0.84)	0.68**	(0.53 - 0.87)	0.99	(0.81 - 1.20)
Second richest	1.12	(0.85 - 1.48)	1.43**	(1.26 - 1.62)	1.20*	(1.01 - 1.43)	1.96**	(1.43 - 2.69)	1.75**	(1.39 - 2.20)
Richest	1.57*	(1.08 - 2.28)	1.84**	(1.57 - 2.16)	1.72**	(1.36 - 2.17)	2.90**	(1.86 - 4.50)	1.74**	(1.33 - 2.28)
Rural residence	1.22	(0.99 - 1.51)	1.35**	(1.23 - 1.49)	1.39**	(1.21 - 1.59)	1.73**	(1.42 - 2.12)	1.00	(0.80 - 1.26)
Had a child who died	0.88	(0.70 - 1.11)	0.84**	(0.76 - 0.92)	0.91	(0.81 - 1.02)	0.76*	(0.61 - 0.95)	0.77*	(0.63 - 0.95)
Has at least one male child	1.16	(0.93 - 1.44)	1.04	(0.92 - 1.17)	0.98	(0.83 - 1.16)	1.27	(0.96 - 1.68)	1.03	(0.81 - 1.29)
Number of children under 5 (biological)	0.88	(0.75 - 1.03)	1.03	(0.98 - 1.09)	1.07	(1.00 - 1.15)	1.08	(0.97 - 1.21)	0.93	(0.85 - 1.03)
Domestic violence justified in some situations (yes)	0.73**	(0.63 - 0.85)	0.72**	(0.67 - 0.78)	0.73**	(0.66 - 0.81)	0.63**	(0.53 - 0.74)	0.83*	(0.71 - 0.96)
Recent child engagement by someone else in HH	0.88	(0.76 - 1.02)	1.14**	(1.06 - 1.23)	1.24**	(1.12 - 1.36)	0.92	(0.77 - 1.10)	1.07	(0.93 - 1.23)
Mother engagement score	1.00	(0.96 - 1.04)	0.99	(0.97 - 1.02)	1.02	(0.98 - 1.06)	0.91**	(0.86 - 0.96)	1.00	(0.95 - 1.04)

*p < .05. **p < .01.

Table A2. A*ssociation between father singing to child in the last 3 days and mothers' life satisfaction, by country and Pakistani province in multilevel logistic regressions.*

	Country				Pakistani Province					
	**Bangladesh** (n=7,754)		**Pakistan** (n= 26,372)		Punjab (n=12,824)		Sindh (n=6,226)		Khyber Pakhtunkhwa (n= 7,322)	
	OR	(95% CI)	OR	(95% CI)	OR	(95% CI)	OR	(95% CI)	OR	(95% CI)
Father sang to child	1.09	(0.86 - 1.38)	1.00	(0.87 - 1.16)	1.18	(0.90 - 1.55)	1.16	(0.91 - 1.48)	0.77*	(0.59 - 1.00)
Child is female	1.20*	(1.00 - 1.43)	0.91**	(0.84 - 0.97)	0.88*	(0.80 - 0.98)	1.00	(0.84 - 1.18)	0.90	(0.79 - 1.02)
Child's birth order	0.87	(0.74 - 1.01)	0.87**	(0.82 - 0.91)	0.89**	(0.83 - 0.96)	0.78**	(0.70 - 0.87)	0.89*	(0.81 - 0.98)
Mother age	1.00	(0.99 - 1.02)	0.99*	(0.98 - 1.00)	0.98**	(0.97 - 0.99)	1.00	(0.99 - 1.02)	0.99	(0.98 - 1.00)
Mother education (ref. = less than primary)										
Primary	1.47**	(1.20 - 1.81)	1.04	(0.93 - 1.15)	1.01	(0.88 - 1.15)	1.08	(0.82 - 1.42)	0.98	(0.78 - 1.24)
Secondary	2.05**	(1.64 - 2.57)	1.16*	(1.03 - 1.31)	1.08	(0.92 - 1.27)	0.95	(0.69 - 1.31)	1.31*	(1.02 - 1.70)
College	2.14**	(1.43 - 3.20)	1.30**	(1.08 - 1.56)	1.18	(0.92 - 1.51)	1.11	(0.70 - 1.77)	1.37	(0.97 - 1.95)
Father education (ref. = less than primary)										
Primary	1.17	(0.98 - 1.40)	1.16**	(1.05 - 1.27)	1.26**	(1.11 - 1.43)	1.15	(0.92 - 1.44)	1.05	(0.85 - 1.29)
Secondary	1.33**	(1.08 - 1.64)	1.29**	(1.18 - 1.41)	1.54**	(1.36 - 1.74)	1.06	(0.85 - 1.33)	1.10	(0.94 - 1.29)
College	2.59**	(1.74 - 3.85)	1.63**	(1.42 - 1.86)	2.11**	(1.70 - 2.60)	1.60**	(1.20 - 2.15)	1.26*	(1.01 - 1.56)
Wealth quintile (ref. = middle)										
Poorest	0.48**	(0.39 - 0.60)	0.56**	(0.50 - 0.62)	0.49**	(0.41 - 0.57)	0.45**	(0.35 - 0.59)	0.80*	(0.65 - 0.98)
Second poorest	0.70**	(0.55 - 0.88)	0.79**	(0.71 - 0.88)	0.72**	(0.62 - 0.84)	0.68**	(0.53 - 0.87)	0.99	(0.81 - 1.20)
Second richest	1.12	(0.85 - 1.48)	1.43**	(1.26 - 1.62)	1.20*	(1.01 - 1.43)	1.97**	(1.44 - 2.71)	1.75**	(1.39 - 2.20)
Richest	1.57*	(1.09 - 2.28)	1.85**	(1.58 - 2.17)	1.72**	(1.37 - 2.17)	2.85**	(1.84 - 4.42)	1.75**	(1.34 - 2.28)
Rural residence	1.22	(0.99 - 1.51)	1.35**	(1.22 - 1.49)	1.39**	(1.21 - 1.59)	1.72**	(1.41 - 2.10)	1.00	(0.80 - 1.26)
Had a child who died	0.88	(0.70 - 1.11)	0.83**	(0.76 - 0.91)	0.91	(0.81 - 1.02)	0.75*	(0.60 - 0.93)	0.77*	(0.63 - 0.94)
Has at least one male child	1.16	(0.94 - 1.44)	1.03	(0.92 - 1.17)	0.98	(0.83 - 1.16)	1.26	(0.96 - 1.67)	1.03	(0.81 - 1.29)
Number of children under 5 (biological)	0.88	(0.75 - 1.03)	1.03	(0.98 - 1.09)	1.07	(1.00 - 1.15)	1.08	(0.96 - 1.20)	0.93	(0.85 - 1.03)
Domestic violence justified in some situations (yes)	0.73**	(0.63 - 0.85)	0.72**	(0.67 - 0.78)	0.73**	(0.66 - 0.81)	0.64**	(0.54 - 0.76)	0.83*	(0.71 - 0.96)
Recent child engagement by someone else in HH	0.89	(0.77 - 1.03)	1.14**	(1.06 - 1.23)	1.24**	(1.12 - 1.36)	0.92	(0.77 - 1.10)	1.07	(0.93 - 1.24)
Mother engagement score	1.01	(0.97 - 1.05)	1.00	(0.98 - 1.03)	1.02	(0.98 - 1.06)	0.95	(0.90 - 1.00)	1.00	(0.96 - 1.05)

*p < .05. **p < .01.

Table A3. A*ssociation between father playing with child in the last 3 days and mothers' life satisfaction, by country and Pakistani province in multilevel logistic regressions.*

	**Country**				**Pakistani Province**					
	**Bangladesh**		**Pakistan**		Punjab		Sindh		Khyber Pakhtunkhwa	
	(n=7,754)		(n= 26,372)		(n=12,824)		(n=6,226)		(n= 7,322)	
	OR	(95% CI)	OR	(95% CI)	OR	(95% CI)	OR	(95% CI)	OR	(95% CI)
Father played with child	0.99	(0.79 - 1.24)	1.02	(0.92 - 1.14)	1.04	(0.88 - 1.22)	0.84	(0.67 - 1.04)	1.18	(0.98 - 1.42)
Child is female	1.20*	(1.00 - 1.42)	0.91**	(0.84 - 0.97)	0.89*	(0.80 - 0.98)	0.99	(0.84 - 1.17)	0.90	(0.79 - 1.03)
Child's birth order	0.87	(0.75 - 1.01)	0.87**	(0.82 - 0.91)	0.89**	(0.83 - 0.96)	0.78**	(0.69 - 0.87)	0.89*	(0.81 - 0.98)
Mother age	1.00	(0.99 - 1.02)	0.99*	(0.98 - 1.00)	0.98**	(0.97 - 0.99)	1.00	(0.99 - 1.02)	0.99	(0.98 - 1.00)
Mother education (ref. = less than primary)										
Primary	1.47**	(1.20 - 1.80)	1.04	(0.93 - 1.15)	1.01	(0.88 - 1.15)	1.07	(0.82 - 1.41)	0.99	(0.78 - 1.25)
Secondary	2.04**	(1.63 - 2.55)	1.16*	(1.03 - 1.31)	1.08	(0.92 - 1.26)	0.95	(0.69 - 1.30)	1.31*	(1.02 - 1.70)
College	2.13**	(1.42 - 3.19)	1.30**	(1.08 - 1.56)	1.18	(0.92 - 1.51)	1.08	(0.68 - 1.73)	1.39	(0.98 - 1.96)
Father education (ref. = less than primary)										
Primary	1.17	(0.98 - 1.40)	1.15**	(1.05 - 1.27)	1.26**	(1.11 - 1.44)	1.16	(0.92 - 1.45)	1.04	(0.85 - 1.28)
Secondary	1.33**	(1.08 - 1.64)	1.29**	(1.18 - 1.41)	1.54**	(1.36 - 1.74)	1.07	(0.85 - 1.33)	1.09	(0.93 - 1.28)
College	2.61**	(1.76 - 3.88)	1.62**	(1.42 - 1.86)	2.11**	(1.71 - 2.61)	1.63**	(1.21 - 2.18)	1.25*	(1.00 - 1.55)
Wealth quintile (ref. = middle)										
Poorest	0.48**	(0.39 - 0.60)	0.56**	(0.50 - 0.62)	0.49**	(0.41 - 0.57)	0.45**	(0.35 - 0.58)	0.80*	(0.66 - 0.98)
Second poorest	0.70**	(0.55 - 0.88)	0.79**	(0.71 - 0.88)	0.72**	(0.62 - 0.84)	0.68**	(0.53 - 0.87)	0.99	(0.81 - 1.20)
Second richest	1.12	(0.85 - 1.48)	1.43**	(1.26 - 1.62)	1.20*	(1.01 - 1.43)	1.96**	(1.43 - 2.69)	1.75**	(1.39 - 2.20)
Richest	1.57*	(1.09 - 2.28)	1.85**	(1.58 - 2.17)	1.72**	(1.37 - 2.17)	2.81**	(1.81 - 4.36)	1.73**	(1.32 - 2.27)
Rural residence	1.22	(0.99 - 1.51)	1.35**	(1.22 - 1.49)	1.39**	(1.21 - 1.59)	1.72**	(1.41 - 2.10)	1.00	(0.80 - 1.25)
Had a child who died	0.88	(0.70 - 1.11)	0.83**	(0.76 - 0.91)	0.91	(0.81 - 1.02)	0.75*	(0.60 - 0.93)	0.77*	(0.63 - 0.95)
Has at least one male child	1.16	(0.94 - 1.44)	1.03	(0.92 - 1.17)	0.98	(0.83 - 1.16)	1.27	(0.96 - 1.68)	1.03	(0.82 - 1.30)
Number of children under 5 (biological)	0.88	(0.75 - 1.03)	1.03	(0.98 - 1.09)	1.07*	(1.00 - 1.15)	1.08	(0.96 - 1.21)	0.93	(0.85 - 1.03)
Domestic violence justified in some situations (yes)	0.73**	(0.63 - 0.85)	0.72**	(0.67 - 0.78)	0.73**	(0.66 - 0.81)	0.64**	(0.54 - 0.76)	0.82*	(0.71 - 0.95)
Recent child engagement by someone else in HH	0.89	(0.77 - 1.03)	1.14**	(1.07 - 1.23)	1.24**	(1.12 - 1.36)	0.92	(0.77 - 1.10)	1.08	(0.94 - 1.24)
Mother engagement score	1.01	(0.97 - 1.05)	1.00	(0.97 - 1.03)	1.02	(0.98 - 1.06)	0.98	(0.93 - 1.03)	0.98	(0.94 - 1.03)

*p < .05. **p < .01.

Table A4. A*ssociation between father taking child outside in the last 3 days and mothers' life satisfaction, by country and Pakistani province in multilevel logistic regressions.*

	**Country**				**Pakistani Province**					
	**Bangladesh**		**Pakistan**		Punjab		Sindh		Khyber Pakhtunkhwa	
	(n=7,754)		(n= 26,372)		(n=12,824)		(n=6,226)		(n= 7,322)	
	OR	(95% CI)	OR	(95% CI)	OR	(95% CI)	OR	(95% CI)	OR	(95% CI)
Father took child outside	1.14	(0.97 - 1.33)	0.99	(0.92 - 1.07)	1.02	(0.92 - 1.13)	0.87	(0.73 - 1.04)	1.05	(0.92 - 1.20)
Child is female	1.20*	(1.01 - 1.43)	0.91**	(0.84 - 0.97)	0.89*	(0.80 - 0.98)	0.99	(0.84 - 1.17)	0.90	(0.79 - 1.03)
Child's birth order	0.87	(0.75 - 1.02)	0.87**	(0.82 - 0.91)	0.89**	(0.83 - 0.96)	0.78**	(0.70 - 0.88)	0.89*	(0.81 - 0.98)
Mother age	1.00	(0.99 - 1.02)	0.99*	(0.98 - 1.00)	0.98**	(0.97 - 0.99)	1.00	(0.99 - 1.02)	0.99	(0.98 - 1.00)
Mother education (ref. = less than primary)										
Primary	1.48**	(1.20 - 1.81)	1.04	(0.93 - 1.15)	1.01	(0.88 - 1.14)	1.07	(0.82 - 1.41)	0.98	(0.78 - 1.24)
Secondary	2.05**	(1.64 - 2.57)	1.16*	(1.03 - 1.31)	1.08	(0.92 - 1.26)	0.96	(0.69 - 1.32)	1.31*	(1.02 - 1.70)
College	2.14**	(1.43 - 3.21)	1.30**	(1.08 - 1.56)	1.18	(0.92 - 1.51)	1.10	(0.69 - 1.75)	1.38	(0.97 - 1.95)
Father education (ref. = less than primary)										
Primary	1.17	(0.98 - 1.40)	1.16**	(1.05 - 1.27)	1.26**	(1.11 - 1.44)	1.15	(0.92 - 1.44)	1.05	(0.85 - 1.28)
Secondary	1.33**	(1.08 - 1.64)	1.29**	(1.18 - 1.41)	1.54**	(1.36 - 1.74)	1.07	(0.86 - 1.33)	1.09	(0.94 - 1.28)
College	2.61**	(1.76 - 3.88)	1.63**	(1.42 - 1.86)	2.11**	(1.71 - 2.61)	1.62**	(1.21 - 2.17)	1.25*	(1.01 - 1.56)
Wealth quintile (ref. = middle)										
Poorest	0.48**	(0.38 - 0.60)	0.56**	(0.50 - 0.62)	0.49**	(0.41 - 0.57)	0.45**	(0.35 - 0.59)	0.80*	(0.66 - 0.98)
Second poorest	0.70**	(0.55 - 0.88)	0.79**	(0.71 - 0.88)	0.72**	(0.62 - 0.84)	0.68**	(0.53 - 0.87)	0.99	(0.81 - 1.20)
Second richest	1.12	(0.85 - 1.48)	1.43**	(1.26 - 1.62)	1.20*	(1.01 - 1.43)	1.96**	(1.43 - 2.69)	1.75**	(1.39 - 2.20)
Richest	1.56*	(1.08 - 2.27)	1.85**	(1.58 - 2.17)	1.72**	(1.37 - 2.17)	2.83**	(1.83 - 4.39)	1.74**	(1.33 - 2.27)
Rural residence	1.22	(0.99 - 1.51)	1.35**	(1.22 - 1.49)	1.39**	(1.21 - 1.59)	1.71**	(1.40 - 2.09)	1.00	(0.80 - 1.26)
Had a child who died	0.87	(0.69 - 1.10)	0.83**	(0.76 - 0.91)	0.91	(0.81 - 1.02)	0.75**	(0.60 - 0.93)	0.77*	(0.63 - 0.95)
Has at least one male child	1.15	(0.93 - 1.43)	1.03	(0.92 - 1.17)	0.98	(0.83 - 1.16)	1.26	(0.95 - 1.66)	1.03	(0.81 - 1.29)
Number of children under 5 (biological)	0.88	(0.75 - 1.03)	1.03	(0.98 - 1.09)	1.07*	(1.00 - 1.15)	1.08	(0.96 - 1.21)	0.93	(0.85 - 1.03)
Domestic violence justified in some situations (yes)	0.73**	(0.63 - 0.85)	0.72**	(0.67 - 0.78)	0.73**	(0.66 - 0.81)	0.64**	(0.54 - 0.76)	0.82*	(0.71 - 0.96)
Recent child engagement by someone else in HH	0.87	(0.75 - 1.01)	1.14**	(1.06 - 1.23)	1.23**	(1.12 - 1.36)	0.96	(0.80 - 1.15)	1.06	(0.92 - 1.22)
Mother engagement score	1.00	(0.97 - 1.04)	1.00	(0.98 - 1.03)	1.02	(0.98 - 1.06)	0.97	(0.93 - 1.02)	0.99	(0.95 - 1.04)

*p < .05. **p < .01.

Table A5. A*ssociation between father telling stories to child in the last 3 days and mothers' life satisfaction, by country and Pakistani province in multilevel logistic regressions.*

	**Country**				**Pakistani Province**					
	**Bangladesh** (n=7,754)		**Pakistan** (n= 26,372)		Punjab (n=12,824)		Sindh (n=6,226)		Khyber Pakhtunkhwa (n= 7,322)	
	OR	(95% CI)	OR	(95% CI)	OR	(95% CI)	OR	(95% CI)	OR	(95% CI)
Father told stories to child	1.19	(0.96 - 1.48)	0.96	(0.84 - 1.09)	0.99	(0.78 - 1.25)	1.25	(0.96 - 1.61)	0.79*	(0.64 - 0.99)
Child is female	1.20*	(1.00 - 1.42)	0.91**	(0.84 - 0.97)	0.88*	(0.80 - 0.98)	1.00	(0.85 - 1.18)	0.90	(0.79 - 1.03)
Child's birth order	0.87	(0.74 - 1.01)	0.87**	(0.82 - 0.91)	0.89**	(0.83 - 0.96)	0.78**	(0.70 - 0.88)	0.89*	(0.81 - 0.98)
Mother age	1.00	(0.99 - 1.02)	0.99*	(0.98 - 1.00)	0.98**	(0.97 - 0.99)	1.00	(0.99 - 1.02)	0.99	(0.98 - 1.00)
Mother education (ref. = less than primary)										
Primary	1.48**	(1.21 - 1.81)	1.04	(0.93 - 1.15)	1.01	(0.88 - 1.15)	1.08	(0.82 - 1.42)	0.99	(0.78 - 1.25)
Secondary	2.06**	(1.65 - 2.58)	1.16*	(1.03 - 1.31)	1.08	(0.92 - 1.26)	0.96	(0.70 - 1.33)	1.32*	(1.02 - 1.70)
College	2.15**	(1.44 - 3.23)	1.30**	(1.08 - 1.56)	1.18	(0.92 - 1.51)	1.11	(0.70 - 1.78)	1.38	(0.98 - 1.96)
Father education (ref. = less than primary)										
Primary	1.17	(0.98 - 1.40)	1.16**	(1.05 - 1.27)	1.26**	(1.11 - 1.44)	1.15	(0.92 - 1.44)	1.05	(0.86 - 1.29)
Secondary	1.32**	(1.08 - 1.63)	1.29**	(1.18 - 1.41)	1.54**	(1.36 - 1.74)	1.06	(0.85 - 1.32)	1.10	(0.94 - 1.29)
College	2.55**	(1.71 - 3.79)	1.63**	(1.43 - 1.86)	2.11**	(1.71 - 2.61)	1.59**	(1.19 - 2.14)	1.28*	(1.03 - 1.59)
Wealth quintile (ref. = middle)										
Poorest	0.48**	(0.39 - 0.60)	0.56**	(0.50 - 0.62)	0.49**	(0.41 - 0.57)	0.45**	(0.35 - 0.59)	0.80*	(0.65 - 0.97)
Second poorest	0.70**	(0.56 - 0.88)	0.79**	(0.71 - 0.88)	0.72**	(0.62 - 0.84)	0.68**	(0.53 - 0.87)	0.99	(0.81 - 1.20)
Second richest	1.12	(0.85 - 1.48)	1.43**	(1.26 - 1.62)	1.20*	(1.01 - 1.43)	1.96**	(1.43 - 2.70)	1.75**	(1.39 - 2.20)
Richest	1.58*	(1.09 - 2.29)	1.85**	(1.58 - 2.17)	1.72**	(1.37 - 2.17)	2.85**	(1.84 - 4.42)	1.75**	(1.34 - 2.29)
Rural residence	1.23	(0.99 - 1.51)	1.35**	(1.22 - 1.49)	1.39**	(1.21 - 1.59)	1.72**	(1.41 - 2.11)	1.00	(0.80 - 1.26)
Had a child who died	0.88	(0.69 - 1.10)	0.83**	(0.76 - 0.91)	0.91	(0.81 - 1.02)	0.75*	(0.61 - 0.93)	0.77*	(0.63 - 0.94)
Has at least one male child	1.16	(0.94 - 1.44)	1.03	(0.92 - 1.17)	0.98	(0.83 - 1.16)	1.26	(0.95 - 1.67)	1.03	(0.82 - 1.30)
Number of children under 5 (biological)	0.88	(0.75 - 1.03)	1.03	(0.98 - 1.09)	1.07	(1.00 - 1.15)	1.08	(0.96 - 1.21)	0.93	(0.85 - 1.03)
Domestic violence justified in some situations (yes)	0.73**	(0.63 - 0.85)	0.73**	(0.67 - 0.78)	0.73**	(0.66 - 0.81)	0.64**	(0.54 - 0.76)	0.83*	(0.71 - 0.96)
Recent child engagement by someone else in HH	0.88	(0.76 - 1.02)	1.14**	(1.07 - 1.23)	1.24**	(1.12 - 1.36)	0.92	(0.77 - 1.09)	1.07	(0.93 - 1.23)
Mother engagement score	1.00	(0.96 - 1.04)	1.00	(0.98 - 1.03)	1.02	(0.99 - 1.06)	0.94*	(0.90 - 0.99)	1.00	(0.96 - 1.05)

*p < .05. **p < .01.

Table A6. A*ssociation between father counting, naming, or drawing things with child in the last 3 days and mothers' life satisfaction, by country and Pakistani province in multilevel logistic regressions.*

	**Country**				**Pakistani Province**					
	**Bangladesh**		**Pakistan**		Punjab		Sindh		Khyber Pakhtunkhwa	
	(n=7,754)		(n= 26,372)		(n=12,824)		(n=6,226)		(n= 7,322)	
	OR	(95% CI)	OR	(95% CI)	OR	(95% CI)	OR	(95% CI)	OR	(95% CI)
Father counted, named, or drew things with child	1.22	(0.97 - 1.52)	1.23*	(1.03 - 1.46)	1.24	(0.93 - 1.65)	1.48*	(1.06 - 2.08)	1.12	(0.83 - 1.51)
Child is female	1.20*	(1.00 - 1.43)	0.91**	(0.84 - 0.97)	0.89*	(0.80 - 0.98)	1.00	(0.85 - 1.18)	0.90	(0.79 - 1.03)
Child's birth order	0.87	(0.75 - 1.01)	0.87**	(0.82 - 0.91)	0.89**	(0.83 - 0.96)	0.78**	(0.70 - 0.88)	0.89*	(0.81 - 0.98)
Mother age	1.00	(0.99 - 1.02)	0.99**	(0.98 - 1.00)	0.98**	(0.97 - 0.99)	1.00	(0.99 - 1.02)	0.99	(0.98 - 1.00)
Mother education (ref. = less than primary)										
Primary	1.48**	(1.21 - 1.81)	1.04	(0.93 - 1.15)	1.01	(0.88 - 1.15)	1.08	(0.82 - 1.42)	0.98	(0.78 - 1.24)
Secondary	2.07**	(1.65 - 2.59)	1.17*	(1.03 - 1.32)	1.08	(0.92 - 1.27)	0.96	(0.70 - 1.33)	1.32*	(1.02 - 1.70)
College	2.15**	(1.44 - 3.23)	1.31**	(1.09 - 1.57)	1.18	(0.92 - 1.52)	1.13	(0.71 - 1.80)	1.38	(0.97 - 1.95)
Father education (ref. = less than primary)										
Primary	1.16	(0.97 - 1.39)	1.15**	(1.05 - 1.27)	1.26**	(1.11 - 1.43)	1.15	(0.92 - 1.44)	1.05	(0.85 - 1.28)
Secondary	1.32**	(1.07 - 1.62)	1.29**	(1.18 - 1.41)	1.54**	(1.36 - 1.74)	1.06	(0.85 - 1.32)	1.09	(0.93 - 1.28)
College	2.54**	(1.71 - 3.78)	1.61**	(1.41 - 1.84)	2.09**	(1.69 - 2.59)	1.58**	(1.18 - 2.12)	1.25*	(1.00 - 1.55)
Wealth quintile (ref. = middle)										
Poorest	0.48**	(0.39 - 0.61)	0.56**	(0.50 - 0.62)	0.49**	(0.41 - 0.57)	0.45**	(0.35 - 0.59)	0.80*	(0.66 - 0.98)
Second poorest	0.70**	(0.55 - 0.88)	0.79**	(0.71 - 0.88)	0.72**	(0.62 - 0.84)	0.69**	(0.53 - 0.88)	0.99	(0.81 - 1.20)
Second richest	1.13	(0.85 - 1.49)	1.42**	(1.26 - 1.62)	1.20*	(1.01 - 1.43)	1.98**	(1.45 - 2.72)	1.74**	(1.38 - 2.19)
Richest	1.57*	(1.08 - 2.28)	1.84**	(1.57 - 2.16)	1.71**	(1.36 - 2.16)	2.87**	(1.85 - 4.46)	1.74**	(1.33 - 2.27)
Rural residence	1.22	(0.99 - 1.51)	1.35**	(1.22 - 1.49)	1.39**	(1.21 - 1.59)	1.72**	(1.40 - 2.10)	1.00	(0.80 - 1.26)
Had a child who died	0.87	(0.69 - 1.10)	0.84**	(0.76 - 0.91)	0.91	(0.81 - 1.02)	0.75*	(0.60 - 0.93)	0.78*	(0.63 - 0.95)
Has at least one male child	1.16	(0.93 - 1.43)	1.04	(0.92 - 1.17)	0.98	(0.83 - 1.16)	1.26	(0.96 - 1.67)	1.03	(0.82 - 1.30)
Number of children under 5 (biological)	0.88	(0.75 - 1.03)	1.03	(0.98 - 1.09)	1.07	(1.00 - 1.15)	1.08	(0.96 - 1.21)	0.93	(0.85 - 1.03)
Domestic violence justified in some situations (yes)	0.74**	(0.63 - 0.86)	0.72**	(0.67 - 0.78)	0.73**	(0.66 - 0.81)	0.63**	(0.54 - 0.75)	0.83*	(0.71 - 0.96)
Recent child engagement by someone else in HH	0.89	(0.77 - 1.02)	1.15**	(1.07 - 1.23)	1.24**	(1.12 - 1.36)	0.93	(0.78 - 1.11)	1.07	(0.93 - 1.23)
Mother engagement score	1.00	(0.96 - 1.04)	0.99	(0.97 - 1.02)	1.02	(0.98 - 1.06)	0.94*	(0.89 - 0.99)	0.99	(0.95 - 1.04)

*p < .05. **p < .01.
