# Supplementary material for: The effect of father-child engagement on maternal life satisfaction in South Asian contexts: evidence from MICS surveys in Pakistan and Bangladesh
Source: Front Sociol. 2026 Jan 7;10:1716756. doi: 10.3389/fsoc.2025.1716756 (PMC12819588; doi:10.3389/fsoc.2025.1716756)
Supplement: Supplementary file 2 [file Table_1.DOCX]

**Appendix**

Table A1. A*ssociation between father reading to child in the last 3 days and mothers’ life satisfaction, by country and Pakistani province in multilevel logistic regressions.*

|  | **Country** | | | | **Pakistani Province** | | | | | |
| --- | --- | --- | --- | --- | --- | --- | --- | --- | --- | --- |
|  | **Bangladesh** | | **Pakistan** | | Punjab | | Sindh | | Khyber Pakhtunkhwa | |
|  | (n=7,754) | | (n= 26,372) | | (n=12,824) | | (n=6,226) | | (n= 7,322) | |
|  | OR | (95% CI) | OR | (95% CI) | OR | (95% CI) | OR | (95% CI) | OR | (95% CI) |
| Father read to child | 1.28* | (1.03 - 1.59) | 1.25** | (1.06 - 1.47) | 1.12 | (0.86 - 1.46) | 2.28** | (1.64 - 3.17) | 0.91 | (0.69 - 1.20) |
|  |  |  |  |  |  |  |  |  |  |  |
| Child is female | 1.20* | (1.00 - 1.43) | 0.91** | (0.85 - 0.97) | 0.89* | (0.80 - 0.98) | 1.00 | (0.84 - 1.18) | 0.90 | (0.79 - 1.03) |
| Child's birth order | 0.87 | (0.75 - 1.01) | 0.87** | (0.82 - 0.91) | 0.89** | (0.83 - 0.96) | 0.78** | (0.70 - 0.88) | 0.89* | (0.81 - 0.98) |
| Mother age | 1.00 | (0.99 - 1.02) | 0.99** | (0.98 - 1.00) | 0.98** | (0.97 - 0.99) | 1.00 | (0.99 - 1.02) | 0.99 | (0.98 - 1.00) |
| Mother education (ref. = less than primary) |  |  |  |  |  |  |  |  |  |  |
| Primary | 1.47** | (1.20 - 1.80) | 1.04 | (0.93 - 1.15) | 1.01 | (0.88 - 1.15) | 1.09 | (0.83 - 1.44) | 0.99 | (0.78 - 1.25) |
| Secondary | 2.05** | (1.64 - 2.57) | 1.17* | (1.03 - 1.32) | 1.08 | (0.92 - 1.26) | 0.97 | (0.70 - 1.34) | 1.32* | (1.02 - 1.70) |
| College | 2.15** | (1.43 - 3.22) | 1.30** | (1.08 - 1.56) | 1.18 | (0.92 - 1.51) | 1.16 | (0.73 - 1.85) | 1.38 | (0.97 - 1.96) |
| Father education (ref. = less than primary) |  |  |  |  |  |  |  |  |  |  |
| Primary | 1.16 | (0.97 - 1.39) | 1.15** | (1.05 - 1.27) | 1.26** | (1.11 - 1.43) | 1.14 | (0.91 - 1.43) | 1.05 | (0.85 - 1.29) |
| Secondary | 1.31* | (1.06 - 1.61) | 1.29** | (1.18 - 1.41) | 1.54** | (1.36 - 1.74) | 1.04 | (0.83 - 1.30) | 1.10 | (0.94 - 1.28) |
| College | 2.49** | (1.67 - 3.71) | 1.61** | (1.41 - 1.84) | 2.10** | (1.70 - 2.60) | 1.54** | (1.15 - 2.06) | 1.26* | (1.02 - 1.57) |
| Wealth quintile (ref. = middle) |  |  |  |  |  |  |  |  |  |  |
| Poorest | 0.48** | (0.39 - 0.60) | 0.56** | (0.50 - 0.62) | 0.49** | (0.41 - 0.57) | 0.45** | (0.35 - 0.58) | 0.80* | (0.65 - 0.97) |
| Second poorest | 0.70** | (0.55 - 0.88) | 0.79** | (0.71 - 0.88) | 0.72** | (0.62 - 0.84) | 0.68** | (0.53 - 0.87) | 0.99 | (0.81 - 1.20) |
| Second richest | 1.12 | (0.85 - 1.48) | 1.43** | (1.26 - 1.62) | 1.20* | (1.01 - 1.43) | 1.96** | (1.43 - 2.69) | 1.75** | (1.39 - 2.20) |
| Richest | 1.57* | (1.08 - 2.28) | 1.84** | (1.57 - 2.16) | 1.72** | (1.36 - 2.17) | 2.90** | (1.86 - 4.50) | 1.74** | (1.33 - 2.28) |
| Rural residence | 1.22 | (0.99 - 1.51) | 1.35** | (1.23 - 1.49) | 1.39** | (1.21 - 1.59) | 1.73** | (1.42 - 2.12) | 1.00 | (0.80 - 1.26) |
| Had a child who died | 0.88 | (0.70 - 1.11) | 0.84** | (0.76 - 0.92) | 0.91 | (0.81 - 1.02) | 0.76* | (0.61 - 0.95) | 0.77* | (0.63 - 0.95) |
| Has at least one male child | 1.16 | (0.93 - 1.44) | 1.04 | (0.92 - 1.17) | 0.98 | (0.83 - 1.16) | 1.27 | (0.96 - 1.68) | 1.03 | (0.81 - 1.29) |
| Number of children under 5 (biological) | 0.88 | (0.75 - 1.03) | 1.03 | (0.98 - 1.09) | 1.07 | (1.00 - 1.15) | 1.08 | (0.97 - 1.21) | 0.93 | (0.85 - 1.03) |
| Domestic violence justified in some situations (yes) | 0.73** | (0.63 - 0.85) | 0.72** | (0.67 - 0.78) | 0.73** | (0.66 - 0.81) | 0.63** | (0.53 - 0.74) | 0.83* | (0.71 - 0.96) |
| Recent child engagement by someone else in HH | 0.88 | (0.76 - 1.02) | 1.14** | (1.06 - 1.23) | 1.24** | (1.12 - 1.36) | 0.92 | (0.77 - 1.10) | 1.07 | (0.93 - 1.23) |
| Mother engagement score | 1.00 | (0.96 - 1.04) | 0.99 | (0.97 - 1.02) | 1.02 | (0.98 - 1.06) | 0.91** | (0.86 - 0.96) | 1.00 | (0.95 - 1.04) |

*p < .05. **p < .01.

Table A2. A*ssociation between father singing to child in the last 3 days and mothers’ life satisfaction, by country and Pakistani province in multilevel logistic regressions.*

|  | **Country** | | | | **Pakistani Province** | | | | | |
| --- | --- | --- | --- | --- | --- | --- | --- | --- | --- | --- |
|  | **Bangladesh** | | **Pakistan** | | Punjab | | Sindh | | Khyber Pakhtunkhwa | |
|  | (n=7,754) | | (n= 26,372) | | (n=12,824) | | (n=6,226) | | (n= 7,322) | |
|  | OR | (95% CI) | OR | (95% CI) | OR | (95% CI) | OR | (95% CI) | OR | (95% CI) |
| Father sang to child | 1.09 | (0.86 - 1.38) | 1.00 | (0.87 - 1.16) | 1.18 | (0.90 - 1.55) | 1.16 | (0.91 - 1.48) | 0.77* | (0.59 - 1.00) |
|  |  |  |  |  |  |  |  |  |  |  |
| Child is female | 1.20* | (1.00 - 1.43) | 0.91** | (0.84 - 0.97) | 0.88* | (0.80 - 0.98) | 1.00 | (0.84 - 1.18) | 0.90 | (0.79 - 1.02) |
| Child's birth order | 0.87 | (0.74 - 1.01) | 0.87** | (0.82 - 0.91) | 0.89** | (0.83 - 0.96) | 0.78** | (0.70 - 0.87) | 0.89* | (0.81 - 0.98) |
| Mother age | 1.00 | (0.99 - 1.02) | 0.99* | (0.98 - 1.00) | 0.98** | (0.97 - 0.99) | 1.00 | (0.99 - 1.02) | 0.99 | (0.98 - 1.00) |
| Mother education (ref. = less than primary) |  |  |  |  |  |  |  |  |  |  |
| Primary | 1.47** | (1.20 - 1.81) | 1.04 | (0.93 - 1.15) | 1.01 | (0.88 - 1.15) | 1.08 | (0.82 - 1.42) | 0.98 | (0.78 - 1.24) |
| Secondary | 2.05** | (1.64 - 2.57) | 1.16* | (1.03 - 1.31) | 1.08 | (0.92 - 1.27) | 0.95 | (0.69 - 1.31) | 1.31* | (1.02 - 1.70) |
| College | 2.14** | (1.43 - 3.20) | 1.30** | (1.08 - 1.56) | 1.18 | (0.92 - 1.51) | 1.11 | (0.70 - 1.77) | 1.37 | (0.97 - 1.95) |
| Father education (ref. = less than primary) |  |  |  |  |  |  |  |  |  |  |
| Primary | 1.17 | (0.98 - 1.40) | 1.16** | (1.05 - 1.27) | 1.26** | (1.11 - 1.43) | 1.15 | (0.92 - 1.44) | 1.05 | (0.85 - 1.29) |
| Secondary | 1.33** | (1.08 - 1.64) | 1.29** | (1.18 - 1.41) | 1.54** | (1.36 - 1.74) | 1.06 | (0.85 - 1.33) | 1.10 | (0.94 - 1.29) |
| College | 2.59** | (1.74 - 3.85) | 1.63** | (1.42 - 1.86) | 2.11** | (1.70 - 2.60) | 1.60** | (1.20 - 2.15) | 1.26* | (1.01 - 1.56) |
| Wealth quintile (ref. = middle) |  |  |  |  |  |  |  |  |  |  |
| Poorest | 0.48** | (0.39 - 0.60) | 0.56** | (0.50 - 0.62) | 0.49** | (0.41 - 0.57) | 0.45** | (0.35 - 0.59) | 0.80* | (0.65 - 0.98) |
| Second poorest | 0.70** | (0.55 - 0.88) | 0.79** | (0.71 - 0.88) | 0.72** | (0.62 - 0.84) | 0.68** | (0.53 - 0.87) | 0.99 | (0.81 - 1.20) |
| Second richest | 1.12 | (0.85 - 1.48) | 1.43** | (1.26 - 1.62) | 1.20* | (1.01 - 1.43) | 1.97** | (1.44 - 2.71) | 1.75** | (1.39 - 2.20) |
| Richest | 1.57* | (1.09 - 2.28) | 1.85** | (1.58 - 2.17) | 1.72** | (1.37 - 2.17) | 2.85** | (1.84 - 4.42) | 1.75** | (1.34 - 2.28) |
| Rural residence | 1.22 | (0.99 - 1.51) | 1.35** | (1.22 - 1.49) | 1.39** | (1.21 - 1.59) | 1.72** | (1.41 - 2.10) | 1.00 | (0.80 - 1.26) |
| Had a child who died | 0.88 | (0.70 - 1.11) | 0.83** | (0.76 - 0.91) | 0.91 | (0.81 - 1.02) | 0.75* | (0.60 - 0.93) | 0.77* | (0.63 - 0.94) |
| Has at least one male child | 1.16 | (0.94 - 1.44) | 1.03 | (0.92 - 1.17) | 0.98 | (0.83 - 1.16) | 1.26 | (0.96 - 1.67) | 1.03 | (0.81 - 1.29) |
| Number of children under 5 (biological) | 0.88 | (0.75 - 1.03) | 1.03 | (0.98 - 1.09) | 1.07 | (1.00 - 1.15) | 1.08 | (0.96 - 1.20) | 0.93 | (0.85 - 1.03) |
| Domestic violence justified in some situations (yes) | 0.73** | (0.63 - 0.85) | 0.72** | (0.67 - 0.78) | 0.73** | (0.66 - 0.81) | 0.64** | (0.54 - 0.76) | 0.83* | (0.71 - 0.96) |
| Recent child engagement by someone else in HH | 0.89 | (0.77 - 1.03) | 1.14** | (1.06 - 1.23) | 1.24** | (1.12 - 1.36) | 0.92 | (0.77 - 1.10) | 1.07 | (0.93 - 1.24) |
| Mother engagement score | 1.01 | (0.97 - 1.05) | 1.00 | (0.98 - 1.03) | 1.02 | (0.98 - 1.06) | 0.95 | (0.90 - 1.00) | 1.00 | (0.96 - 1.05) |

*p < .05. **p < .01.

Table A3. A*ssociation between father playing with child in the last 3 days and mothers’ life satisfaction, by country and Pakistani province in multilevel logistic regressions.*

|  | **Country** | | | | **Pakistani Province** | | | | | |
| --- | --- | --- | --- | --- | --- | --- | --- | --- | --- | --- |
|  | **Bangladesh** | | **Pakistan** | | Punjab | | Sindh | | Khyber Pakhtunkhwa | |
|  | (n=7,754) | | (n= 26,372) | | (n=12,824) | | (n=6,226) | | (n= 7,322) | |
|  | OR | (95% CI) | OR | (95% CI) | OR | (95% CI) | OR | (95% CI) | OR | (95% CI) |
| Father played with child | 0.99 | (0.79 - 1.24) | 1.02 | (0.92 - 1.14) | 1.04 | (0.88 - 1.22) | 0.84 | (0.67 - 1.04) | 1.18 | (0.98 - 1.42) |
| Child is female | 1.20* | (1.00 - 1.42) | 0.91** | (0.84 - 0.97) | 0.89* | (0.80 - 0.98) | 0.99 | (0.84 - 1.17) | 0.90 | (0.79 - 1.03) |
| Child's birth order | 0.87 | (0.75 - 1.01) | 0.87** | (0.82 - 0.91) | 0.89** | (0.83 - 0.96) | 0.78** | (0.69 - 0.87) | 0.89* | (0.81 - 0.98) |
| Mother age | 1.00 | (0.99 - 1.02) | 0.99* | (0.98 - 1.00) | 0.98** | (0.97 - 0.99) | 1.00 | (0.99 - 1.02) | 0.99 | (0.98 - 1.00) |
| Mother education (ref. = less than primary) |  |  |  |  |  |  |  |  |  |  |
| Primary | 1.47** | (1.20 - 1.80) | 1.04 | (0.93 - 1.15) | 1.01 | (0.88 - 1.15) | 1.07 | (0.82 - 1.41) | 0.99 | (0.78 - 1.25) |
| Secondary | 2.04** | (1.63 - 2.55) | 1.16* | (1.03 - 1.31) | 1.08 | (0.92 - 1.26) | 0.95 | (0.69 - 1.30) | 1.31* | (1.02 - 1.70) |
| College | 2.13** | (1.42 - 3.19) | 1.30** | (1.08 - 1.56) | 1.18 | (0.92 - 1.51) | 1.08 | (0.68 - 1.73) | 1.39 | (0.98 - 1.96) |
| Father education (ref. = less than primary) |  |  |  |  |  |  |  |  |  |  |
| Primary | 1.17 | (0.98 - 1.40) | 1.15** | (1.05 - 1.27) | 1.26** | (1.11 - 1.44) | 1.16 | (0.92 - 1.45) | 1.04 | (0.85 - 1.28) |
| Secondary | 1.33** | (1.08 - 1.64) | 1.29** | (1.18 - 1.41) | 1.54** | (1.36 - 1.74) | 1.07 | (0.85 - 1.33) | 1.09 | (0.93 - 1.28) |
| College | 2.61** | (1.76 - 3.88) | 1.62** | (1.42 - 1.86) | 2.11** | (1.71 - 2.61) | 1.63** | (1.21 - 2.18) | 1.25* | (1.00 - 1.55) |
| Wealth quintile (ref. = middle) |  |  |  |  |  |  |  |  |  |  |
| Poorest | 0.48** | (0.39 - 0.60) | 0.56** | (0.50 - 0.62) | 0.49** | (0.41 - 0.57) | 0.45** | (0.35 - 0.58) | 0.80* | (0.66 - 0.98) |
| Second poorest | 0.70** | (0.55 - 0.88) | 0.79** | (0.71 - 0.88) | 0.72** | (0.62 - 0.84) | 0.68** | (0.53 - 0.87) | 0.99 | (0.81 - 1.20) |
| Second richest | 1.12 | (0.85 - 1.48) | 1.43** | (1.26 - 1.62) | 1.20* | (1.01 - 1.43) | 1.96** | (1.43 - 2.69) | 1.75** | (1.39 - 2.20) |
| Richest | 1.57* | (1.09 - 2.28) | 1.85** | (1.58 - 2.17) | 1.72** | (1.37 - 2.17) | 2.81** | (1.81 - 4.36) | 1.73** | (1.32 - 2.27) |
| Rural residence | 1.22 | (0.99 - 1.51) | 1.35** | (1.22 - 1.49) | 1.39** | (1.21 - 1.59) | 1.72** | (1.41 - 2.10) | 1.00 | (0.80 - 1.25) |
| Had a child who died | 0.88 | (0.70 - 1.11) | 0.83** | (0.76 - 0.91) | 0.91 | (0.81 - 1.02) | 0.75* | (0.60 - 0.93) | 0.77* | (0.63 - 0.95) |
| Has at least one male child | 1.16 | (0.94 - 1.44) | 1.03 | (0.92 - 1.17) | 0.98 | (0.83 - 1.16) | 1.27 | (0.96 - 1.68) | 1.03 | (0.82 - 1.30) |
| Number of children under 5 (biological) | 0.88 | (0.75 - 1.03) | 1.03 | (0.98 - 1.09) | 1.07* | (1.00 - 1.15) | 1.08 | (0.96 - 1.21) | 0.93 | (0.85 - 1.03) |
| Domestic violence justified in some situations (yes) | 0.73** | (0.63 - 0.85) | 0.72** | (0.67 - 0.78) | 0.73** | (0.66 - 0.81) | 0.64** | (0.54 - 0.76) | 0.82* | (0.71 - 0.95) |
| Recent child engagement by someone else in HH | 0.89 | (0.77 - 1.03) | 1.14** | (1.07 - 1.23) | 1.24** | (1.12 - 1.36) | 0.92 | (0.77 - 1.10) | 1.08 | (0.94 - 1.24) |
| Mother engagement score | 1.01 | (0.97 - 1.05) | 1.00 | (0.97 - 1.03) | 1.02 | (0.98 - 1.06) | 0.98 | (0.93 - 1.03) | 0.98 | (0.94 - 1.03) |

*p < .05. **p < .01.

|  | **Country** | | | | **Pakistani Province** | | | | | |
| --- | --- | --- | --- | --- | --- | --- | --- | --- | --- | --- |
|  | **Bangladesh** | | **Pakistan** | | Punjab | | Sindh | | Khyber Pakhtunkhwa | |
|  | (n=7,754) | | (n= 26,372) | | (n=12,824) | | (n=6,226) | | (n= 7,322) | |
|  | OR | (95% CI) | OR | (95% CI) | OR | (95% CI) | OR | (95% CI) | OR | (95% CI) |
| Father took child outside | 1.14 | (0.97 - 1.33) | 0.99 | (0.92 - 1.07) | 1.02 | (0.92 - 1.13) | 0.87 | (0.73 - 1.04) | 1.05 | (0.92 - 1.20) |
| Child is female | 1.20* | (1.01 - 1.43) | 0.91** | (0.84 - 0.97) | 0.89* | (0.80 - 0.98) | 0.99 | (0.84 - 1.17) | 0.90 | (0.79 - 1.03) |
| Child's birth order | 0.87 | (0.75 - 1.02) | 0.87** | (0.82 - 0.91) | 0.89** | (0.83 - 0.96) | 0.78** | (0.70 - 0.88) | 0.89* | (0.81 - 0.98) |
| Mother age | 1.00 | (0.99 - 1.02) | 0.99* | (0.98 - 1.00) | 0.98** | (0.97 - 0.99) | 1.00 | (0.99 - 1.02) | 0.99 | (0.98 - 1.00) |
| Mother education (ref. = less than primary) |  |  |  |  |  |  |  |  |  |  |
| Primary | 1.48** | (1.20 - 1.81) | 1.04 | (0.93 - 1.15) | 1.01 | (0.88 - 1.14) | 1.07 | (0.82 - 1.41) | 0.98 | (0.78 - 1.24) |
| Secondary | 2.05** | (1.64 - 2.57) | 1.16* | (1.03 - 1.31) | 1.08 | (0.92 - 1.26) | 0.96 | (0.69 - 1.32) | 1.31* | (1.02 - 1.70) |
| College | 2.14** | (1.43 - 3.21) | 1.30** | (1.08 - 1.56) | 1.18 | (0.92 - 1.51) | 1.10 | (0.69 - 1.75) | 1.38 | (0.97 - 1.95) |
| Father education (ref. = less than primary) |  |  |  |  |  |  |  |  |  |  |
| Primary | 1.17 | (0.98 - 1.40) | 1.16** | (1.05 - 1.27) | 1.26** | (1.11 - 1.44) | 1.15 | (0.92 - 1.44) | 1.05 | (0.85 - 1.28) |
| Secondary | 1.33** | (1.08 - 1.64) | 1.29** | (1.18 - 1.41) | 1.54** | (1.36 - 1.74) | 1.07 | (0.86 - 1.33) | 1.09 | (0.94 - 1.28) |
| College | 2.61** | (1.76 - 3.88) | 1.63** | (1.42 - 1.86) | 2.11** | (1.71 - 2.61) | 1.62** | (1.21 - 2.17) | 1.25* | (1.01 - 1.56) |
| Wealth quintile (ref. = middle) |  |  |  |  |  |  |  |  |  |  |
| Poorest | 0.48** | (0.38 - 0.60) | 0.56** | (0.50 - 0.62) | 0.49** | (0.41 - 0.57) | 0.45** | (0.35 - 0.59) | 0.80* | (0.66 - 0.98) |
| Second poorest | 0.70** | (0.55 - 0.88) | 0.79** | (0.71 - 0.88) | 0.72** | (0.62 - 0.84) | 0.68** | (0.53 - 0.87) | 0.99 | (0.81 - 1.20) |
| Second richest | 1.12 | (0.85 - 1.48) | 1.43** | (1.26 - 1.62) | 1.20* | (1.01 - 1.43) | 1.96** | (1.43 - 2.69) | 1.75** | (1.39 - 2.20) |
| Richest | 1.56* | (1.08 - 2.27) | 1.85** | (1.58 - 2.17) | 1.72** | (1.37 - 2.17) | 2.83** | (1.83 - 4.39) | 1.74** | (1.33 - 2.27) |
| Rural residence | 1.22 | (0.99 - 1.51) | 1.35** | (1.22 - 1.49) | 1.39** | (1.21 - 1.59) | 1.71** | (1.40 - 2.09) | 1.00 | (0.80 - 1.26) |
| Had a child who died | 0.87 | (0.69 - 1.10) | 0.83** | (0.76 - 0.91) | 0.91 | (0.81 - 1.02) | 0.75** | (0.60 - 0.93) | 0.77* | (0.63 - 0.95) |
| Has at least one male child | 1.15 | (0.93 - 1.43) | 1.03 | (0.92 - 1.17) | 0.98 | (0.83 - 1.16) | 1.26 | (0.95 - 1.66) | 1.03 | (0.81 - 1.29) |
| Number of children under 5 (biological) | 0.88 | (0.75 - 1.03) | 1.03 | (0.98 - 1.09) | 1.07* | (1.00 - 1.15) | 1.08 | (0.96 - 1.21) | 0.93 | (0.85 - 1.03) |
| Domestic violence justified in some situations (yes) | 0.73** | (0.63 - 0.85) | 0.72** | (0.67 - 0.78) | 0.73** | (0.66 - 0.81) | 0.64** | (0.54 - 0.76) | 0.82* | (0.71 - 0.96) |
| Recent child engagement by someone else in HH | 0.87 | (0.75 - 1.01) | 1.14** | (1.06 - 1.23) | 1.23** | (1.12 - 1.36) | 0.96 | (0.80 - 1.15) | 1.06 | (0.92 - 1.22) |
| Mother engagement score | 1.00 | (0.97 - 1.04) | 1.00 | (0.98 - 1.03) | 1.02 | (0.98 - 1.06) | 0.97 | (0.93 - 1.02) | 0.99 | (0.95 - 1.04) |

Table A4. A*ssociation between father taking child outside in the last 3 days and mothers’ life satisfaction, by country and Pakistani province in multilevel logistic regressions.*

*p < .05. **p < .01.

|  | **Country** | | | | **Pakistani Province** | | | | | |
| --- | --- | --- | --- | --- | --- | --- | --- | --- | --- | --- |
|  | **Bangladesh** | | **Pakistan** | | Punjab | | Sindh | | Khyber Pakhtunkhwa | |
|  | (n=7,754) | | (n= 26,372) | | (n=12,824) | | (n=6,226) | | (n= 7,322) | |
|  | OR | (95% CI) | OR | (95% CI) | OR | (95% CI) | OR | (95% CI) | OR | (95% CI) |
| Father told stories to child | 1.19 | (0.96 - 1.48) | 0.96 | (0.84 - 1.09) | 0.99 | (0.78 - 1.25) | 1.25 | (0.96 - 1.61) | 0.79* | (0.64 - 0.99) |
| Child is female | 1.20* | (1.00 - 1.42) | 0.91** | (0.84 - 0.97) | 0.88* | (0.80 - 0.98) | 1.00 | (0.85 - 1.18) | 0.90 | (0.79 - 1.03) |
| Child's birth order | 0.87 | (0.74 - 1.01) | 0.87** | (0.82 - 0.91) | 0.89** | (0.83 - 0.96) | 0.78** | (0.70 - 0.88) | 0.89* | (0.81 - 0.98) |
| Mother age | 1.00 | (0.99 - 1.02) | 0.99* | (0.98 - 1.00) | 0.98** | (0.97 - 0.99) | 1.00 | (0.99 - 1.02) | 0.99 | (0.98 - 1.00) |
| Mother education (ref. = less than primary) |  |  |  |  |  |  |  |  |  |  |
| Primary | 1.48** | (1.21 - 1.81) | 1.04 | (0.93 - 1.15) | 1.01 | (0.88 - 1.15) | 1.08 | (0.82 - 1.42) | 0.99 | (0.78 - 1.25) |
| Secondary | 2.06** | (1.65 - 2.58) | 1.16* | (1.03 - 1.31) | 1.08 | (0.92 - 1.26) | 0.96 | (0.70 - 1.33) | 1.32* | (1.02 - 1.70) |
| College | 2.15** | (1.44 - 3.23) | 1.30** | (1.08 - 1.56) | 1.18 | (0.92 - 1.51) | 1.11 | (0.70 - 1.78) | 1.38 | (0.98 - 1.96) |
| Father education (ref. = less than primary) |  |  |  |  |  |  |  |  |  |  |
| Primary | 1.17 | (0.98 - 1.40) | 1.16** | (1.05 - 1.27) | 1.26** | (1.11 - 1.44) | 1.15 | (0.92 - 1.44) | 1.05 | (0.86 - 1.29) |
| Secondary | 1.32** | (1.08 - 1.63) | 1.29** | (1.18 - 1.41) | 1.54** | (1.36 - 1.74) | 1.06 | (0.85 - 1.32) | 1.10 | (0.94 - 1.29) |
| College | 2.55** | (1.71 - 3.79) | 1.63** | (1.43 - 1.86) | 2.11** | (1.71 - 2.61) | 1.59** | (1.19 - 2.14) | 1.28* | (1.03 - 1.59) |
| Wealth quintile (ref. = middle) |  |  |  |  |  |  |  |  |  |  |
| Poorest | 0.48** | (0.39 - 0.60) | 0.56** | (0.50 - 0.62) | 0.49** | (0.41 - 0.57) | 0.45** | (0.35 - 0.59) | 0.80* | (0.65 - 0.97) |
| Second poorest | 0.70** | (0.56 - 0.88) | 0.79** | (0.71 - 0.88) | 0.72** | (0.62 - 0.84) | 0.68** | (0.53 - 0.87) | 0.99 | (0.81 - 1.20) |
| Second richest | 1.12 | (0.85 - 1.48) | 1.43** | (1.26 - 1.62) | 1.20* | (1.01 - 1.43) | 1.96** | (1.43 - 2.70) | 1.75** | (1.39 - 2.20) |
| Richest | 1.58* | (1.09 - 2.29) | 1.85** | (1.58 - 2.17) | 1.72** | (1.37 - 2.17) | 2.85** | (1.84 - 4.42) | 1.75** | (1.34 - 2.29) |
| Rural residence | 1.23 | (0.99 - 1.51) | 1.35** | (1.22 - 1.49) | 1.39** | (1.21 - 1.59) | 1.72** | (1.41 - 2.11) | 1.00 | (0.80 - 1.26) |
| Had a child who died | 0.88 | (0.69 - 1.10) | 0.83** | (0.76 - 0.91) | 0.91 | (0.81 - 1.02) | 0.75* | (0.61 - 0.93) | 0.77* | (0.63 - 0.94) |
| Has at least one male child | 1.16 | (0.94 - 1.44) | 1.03 | (0.92 - 1.17) | 0.98 | (0.83 - 1.16) | 1.26 | (0.95 - 1.67) | 1.03 | (0.82 - 1.30) |
| Number of children under 5 (biological) | 0.88 | (0.75 - 1.03) | 1.03 | (0.98 - 1.09) | 1.07 | (1.00 - 1.15) | 1.08 | (0.96 - 1.21) | 0.93 | (0.85 - 1.03) |
| Domestic violence justified in some situations (yes) | 0.73** | (0.63 - 0.85) | 0.73** | (0.67 - 0.78) | 0.73** | (0.66 - 0.81) | 0.64** | (0.54 - 0.76) | 0.83* | (0.71 - 0.96) |
| Recent child engagement by someone else in HH | 0.88 | (0.76 - 1.02) | 1.14** | (1.07 - 1.23) | 1.24** | (1.12 - 1.36) | 0.92 | (0.77 - 1.09) | 1.07 | (0.93 - 1.23) |
| Mother engagement score | 1.00 | (0.96 - 1.04) | 1.00 | (0.98 - 1.03) | 1.02 | (0.99 - 1.06) | 0.94* | (0.90 - 0.99) | 1.00 | (0.96 - 1.05) |

Table A5. A*ssociation between father telling stories to child in the last 3 days and mothers’ life satisfaction, by country and Pakistani province in multilevel logistic regressions.*

*p < .05. **p < .01.

Table A6. A*ssociation between father counting, naming, or drawing things with child in the last 3 days and mothers’ life satisfaction, by country and Pakistani province in multilevel logistic regressions.*

|  | **Country** | | | | **Pakistani Province** | | | | | |
| --- | --- | --- | --- | --- | --- | --- | --- | --- | --- | --- |
|  | **Bangladesh** | | **Pakistan** | | Punjab | | Sindh | | Khyber Pakhtunkhwa | |
|  | (n=7,754) | | (n= 26,372) | | (n=12,824) | | (n=6,226) | | (n= 7,322) | |
|  | OR | (95% CI) | OR | (95% CI) | OR | (95% CI) | OR | (95% CI) | OR | (95% CI) |
| Father counted, named,  or drew things with child | 1.22 | (0.97 - 1.52) | 1.23* | (1.03 - 1.46) | 1.24 | (0.93 - 1.65) | 1.48* | (1.06 - 2.08) | 1.12 | (0.83 - 1.51) |
| Child is female | 1.20* | (1.00 - 1.43) | 0.91** | (0.84 - 0.97) | 0.89* | (0.80 - 0.98) | 1.00 | (0.85 - 1.18) | 0.90 | (0.79 - 1.03) |
| Child's birth order | 0.87 | (0.75 - 1.01) | 0.87** | (0.82 - 0.91) | 0.89** | (0.83 - 0.96) | 0.78** | (0.70 - 0.88) | 0.89* | (0.81 - 0.98) |
| Mother age | 1.00 | (0.99 - 1.02) | 0.99** | (0.98 - 1.00) | 0.98** | (0.97 - 0.99) | 1.00 | (0.99 - 1.02) | 0.99 | (0.98 - 1.00) |
| Mother education (ref. = less than primary) |  |  |  |  |  |  |  |  |  |  |
| Primary | 1.48** | (1.21 - 1.81) | 1.04 | (0.93 - 1.15) | 1.01 | (0.88 - 1.15) | 1.08 | (0.82 - 1.42) | 0.98 | (0.78 - 1.24) |
| Secondary | 2.07** | (1.65 - 2.59) | 1.17* | (1.03 - 1.32) | 1.08 | (0.92 - 1.27) | 0.96 | (0.70 - 1.33) | 1.32* | (1.02 - 1.70) |
| College | 2.15** | (1.44 - 3.23) | 1.31** | (1.09 - 1.57) | 1.18 | (0.92 - 1.52) | 1.13 | (0.71 - 1.80) | 1.38 | (0.97 - 1.95) |
| Father education (ref. = less than primary) |  |  |  |  |  |  |  |  |  |  |
| Primary | 1.16 | (0.97 - 1.39) | 1.15** | (1.05 - 1.27) | 1.26** | (1.11 - 1.43) | 1.15 | (0.92 - 1.44) | 1.05 | (0.85 - 1.28) |
| Secondary | 1.32** | (1.07 - 1.62) | 1.29** | (1.18 - 1.41) | 1.54** | (1.36 - 1.74) | 1.06 | (0.85 - 1.32) | 1.09 | (0.93 - 1.28) |
| College | 2.54** | (1.71 - 3.78) | 1.61** | (1.41 - 1.84) | 2.09** | (1.69 - 2.59) | 1.58** | (1.18 - 2.12) | 1.25* | (1.00 - 1.55) |
| Wealth quintile (ref. = middle) |  |  |  |  |  |  |  |  |  |  |
| Poorest | 0.48** | (0.39 - 0.61) | 0.56** | (0.50 - 0.62) | 0.49** | (0.41 - 0.57) | 0.45** | (0.35 - 0.59) | 0.80* | (0.66 - 0.98) |
| Second poorest | 0.70** | (0.55 - 0.88) | 0.79** | (0.71 - 0.88) | 0.72** | (0.62 - 0.84) | 0.69** | (0.53 - 0.88) | 0.99 | (0.81 - 1.20) |
| Second richest | 1.13 | (0.85 - 1.49) | 1.42** | (1.26 - 1.62) | 1.20* | (1.01 - 1.43) | 1.98** | (1.45 - 2.72) | 1.74** | (1.38 - 2.19) |
| Richest | 1.57* | (1.08 - 2.28) | 1.84** | (1.57 - 2.16) | 1.71** | (1.36 - 2.16) | 2.87** | (1.85 - 4.46) | 1.74** | (1.33 - 2.27) |
| Rural residence | 1.22 | (0.99 - 1.51) | 1.35** | (1.22 - 1.49) | 1.39** | (1.21 - 1.59) | 1.72** | (1.40 - 2.10) | 1.00 | (0.80 - 1.26) |
| Had a child who died | 0.87 | (0.69 - 1.10) | 0.84** | (0.76 - 0.91) | 0.91 | (0.81 - 1.02) | 0.75* | (0.60 - 0.93) | 0.78* | (0.63 - 0.95) |
| Has at least one male child | 1.16 | (0.93 - 1.43) | 1.04 | (0.92 - 1.17) | 0.98 | (0.83 - 1.16) | 1.26 | (0.96 - 1.67) | 1.03 | (0.82 - 1.30) |
| Number of children under 5 (biological) | 0.88 | (0.75 - 1.03) | 1.03 | (0.98 - 1.09) | 1.07 | (1.00 - 1.15) | 1.08 | (0.96 - 1.21) | 0.93 | (0.85 - 1.03) |
| Domestic violence justified in some situations (yes) | 0.74** | (0.63 - 0.86) | 0.72** | (0.67 - 0.78) | 0.73** | (0.66 - 0.81) | 0.63** | (0.54 - 0.75) | 0.83* | (0.71 - 0.96) |
| Recent child engagement by someone else in HH | 0.89 | (0.77 - 1.02) | 1.15** | (1.07 - 1.23) | 1.24** | (1.12 - 1.36) | 0.93 | (0.78 - 1.11) | 1.07 | (0.93 - 1.23) |
| Mother engagement score | 1.00 | (0.96 - 1.04) | 0.99 | (0.97 - 1.02) | 1.02 | (0.98 - 1.06) | 0.94* | (0.89 - 0.99) | 0.99 | (0.95 - 1.04) |

*p < .05. **p < .01.
